# Stroma-associated master regulators of molecular subtypes predict patient prognosis in ovarian cancer

**DOI:** 10.1038/srep16066

**Published:** 2015-11-04

**Authors:** Shengzhe Zhang, Ying Jing, Meiying Zhang, Zhenfeng Zhang, Pengfei Ma, Huixin Peng, Kaixuan Shi, Wei-Qiang Gao, Guanglei Zhuang

**Affiliations:** 1 State Key Laboratory of Oncogenes and Related Genes, Renji-Med X Clinical Stem Cell Research Center, Ren Ji Hospital, School of Medicine, Shanghai Jiao Tong University, Shanghai, China; 2 School of Biomedical Engineering & Med-X Research Institute, Shanghai Jiao Tong University, Shanghai, China; 3 Department of Obstetrics and Gynecology, Ren Ji Hospital, School of Medicine, Shanghai Jiao Tong University, Shanghai, China; 4 Shanghai Key Laboratory of Gynecologic Oncology, Ren Ji Hospital, School of Medicine, Shanghai Jiao Tong University, Shanghai, China; 5 State Key Laboratory of Oncogenes and Related Genes, Shanghai Cancer Institute, Ren Ji Hospital, School of Medicine, Shanghai Jiao Tong University, Shanghai, China

## Abstract

High-grade serous ovarian carcinoma (HGS-OvCa) has the lowest survival rate among all gynecologic cancers and is hallmarked by a high degree of heterogeneity. The Cancer Genome Atlas network has described a gene expression-based molecular classification of HGS-OvCa into Differentiated, Mesenchymal, Immunoreactive and Proliferative subtypes. However, the biological underpinnings and regulatory mechanisms underlying the distinct molecular subtypes are largely unknown. Here we showed that tumor-infiltrating stromal cells significantly contributed to the assignments of Mesenchymal and Immunoreactive clusters. Using reverse engineering and an unbiased interrogation of subtype regulatory networks, we identified the transcriptional modules containing master regulators that drive gene expression of Mesenchymal and Immunoreactive HGS-OvCa. Mesenchymal master regulators were associated with poor prognosis, while Immunoreactive master regulators positively correlated with overall survival. Meta-analysis of 749 HGS-OvCa expression profiles confirmed that master regulators as a prognostic signature were able to predict patient outcome. Our data unraveled master regulatory programs of HGS-OvCa subtypes with prognostic and potentially therapeutic relevance, and suggested that the unique transcriptional and clinical characteristics of ovarian Mesenchymal and Immunoreactive subtypes could be, at least partially, ascribed to tumor microenvironment.

High-grade serous ovarian carcinoma (HGS-OvCa) is the most lethal gynecological cancer and represents a clinically heterogeneous disease^1^,^2^,^3^. For example, essentially all patients diagnosed with advanced disease undergo very similar standard treatment, which is aggressive surgical debulking followed by multi-cycles of platinum-based combination chemotherapy^4^. However, approximately 30% of cases exhibit intrinsic chemoresistance and gain little or no benefit. Additionally, a large percentage of chemosensitive patients develop acquired resistance and eventually relapse within various time windows^5^,^6^. Therefore, it is important to leverage novel prognostic tools to stratify seemingly identical patients and redirect them to more precise therapies that may be potentially efficacious.

To complement conventional histopathology, major efforts have recently been focused on the molecular classifications enabled by large-scale global gene expression profiling studies. Several groups have used microarray-based gene expression datasets to retrospectively classify HGS-OvCa patients into prognostic and/or molecular subtypes^7^. Using k-means clustering, Tothill *et al.* reported six molecular subtypes in 285 serous and endometrioid tumors, and defined a poor prognosis subtype by a reactive stroma gene expression signature^8^. Tan *et al.* presented a meta-analysis of epithelial ovarian cancer and identified five distinct subgroups, which exhibited significantly different patient outcome^9^. Nevertheless, these classification schemes have not yet achieved widespread application, partly due to the lack of imperative understanding of biologic rationale that determines the transcriptional and clinical characteristics of diverse subtypes.

Recently, the Cancer Genome Atlas (TCGA) network identified four HGS-OvCa subtypes^10^, namely Differentiated, Mesenchymal, Immunoreactive and Proliferative, which were subsequently validated in an independent patient cohort (Mayo Clinic cohort)^11^. Surprisingly, however, survival time did not differ significantly for the transcriptional subtypes in the TCGA HGS-OvCa dataset^10^, in contrast to the clinical relevance of molecular classifiers evident in other cancers^12^,^13^,^14^. Counterintuitively, a statistically significant difference in patient survival was observed in the Mayo Clinic cohort, i.e. the Immunoreactive subtype had the longest survival time, while the Mesenchymal subtype had the shortest. These inconsistent findings necessitate further prudent investigations before employing the TCGA subtyping in patient stratification.

We reasoned that a more thorough understanding of the biological and regulatory mechanisms underlying the distinct subtypes might facilitate the development of novel prognostic signatures and subtype-specific therapeutic strategies in HGS-OvCa. For example, numerous studies have implicated tumor-associated stroma in tumor progression and patient prognosis^15^,^16^,^17^. Interestingly, it has been recently discovered that stromal genes significantly contributed to the stem/serrated/mesenchymal transcriptional subtype in colorectal cancer^18^,^19^. Although the Mesenchymal and Immunoreactive subtypes of ovarian cancer are known to contain infiltrating stromal cells and lymphocytes, respectively, it remains to be determined whether and to what extent tumor microenvironment influences the assignment of transcriptional subtypes. In this study, we designed an analytical approach to delineate the cellular and molecular underpinnings of HGS-OvCa subtypes, with a specific focus on the involvement of tumor stromal constituents.

## Results

### The TCGA subtypes are not associated with patient prognosis

**Figure 1:**
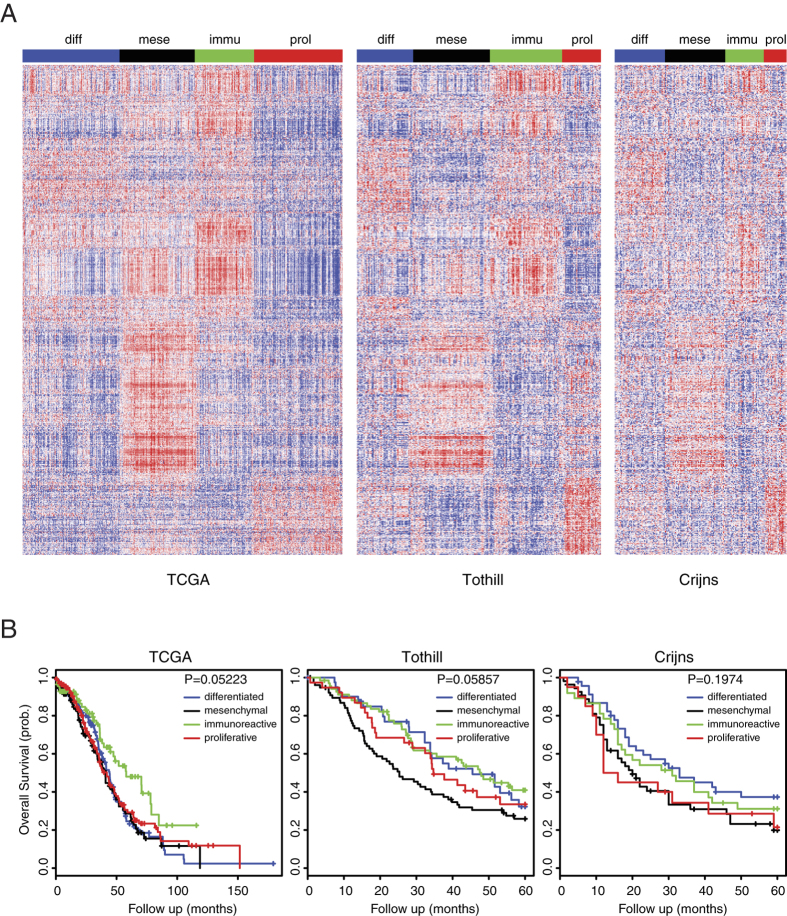
The TCGA subtypes are not associated with patient prognosis. (**A**) Tumors from TCGA, Tothill and Crijns datasets were separated into four clusters on the basis of gene expression. (**B**) Kaplan Meier curves for four molecular subtypes in the TCGA, Tothill and Crijns datasets.

Both non-negative matrix factorization (NMF) method (Supplementary Figure S1) and k-means clustering algorithm (Supplementary Figure S2) yielded four robust high-consensus molecular subtypes in the TCGA dataset, thus verifying previous classifications^10^. We calculated silhouette width^20^ to identify samples most representative of each clusters and obtained a ‘core’ set of 388 tumors (Supplementary Figure S3). Subsequently, we derived a 749-gene classifier (Supplementary Table S1) with the lowest prediction error using significance analysis of microarrays (SAM)^21^, followed by prediction analysis for microarrays (PAM)^22^. We applied the 749-gene signature and NMF consensus clustering in two independent HGS-OvCa gene expression profiles (Tothill and Crijns)^8^,^23^, and validated the four molecular subtypes (Fig. 1A; Supplementary Figure S4-5). However, in all three datasets, the HGS-OvCa molecular subtypes were not prognostically relevant (Fig. 1B). These unexpected results prompted us to further investigate the cellular and molecular determinants of HGS-OvCa clusters.

### Tumor-associated stromal content contributes to defining Mesenchymal and Immunoreactive subtypes

**Figure 2:**
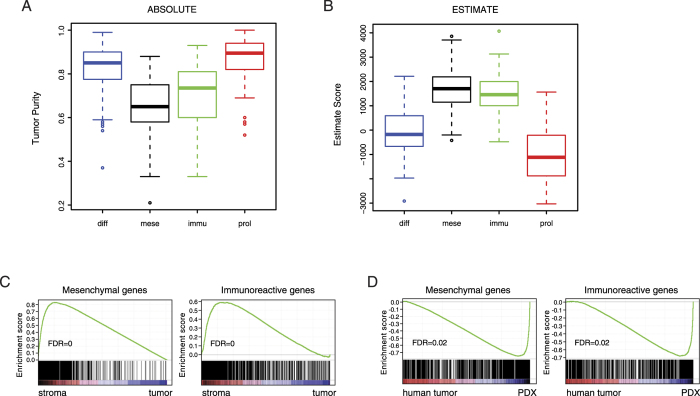
Tumor-associated stromal content contributes to defining Mesenchymal and Immunoreactive subtypes. (**A**) Tumor purity estimated by ABSOLUTE analysis for four molecular subtypes of TCGA samples. (**B**) ESTIMATE scores for four molecular subtypes of TCGA samples. (**C**) GSEA for upregulation of Mesenchymal and Immunoreactive genes in microdissected tumor stroma versus epithelial tissues. (**D**) GSEA for downregulation of Mesenchymal and Immunoreactive genes in patient-derived xenografts (PDX) versus matched primary tumors.

The four TCGA subtypes were initially termed Differentiated, Mesenchymal, Immunoreactive and Proliferative on the basis of expressed genes in the clusters^10^. For example, the Mesenchymal subtype was defined by high expression of FAP, fibronectin and collagens, whereas chemokine ligands (CXCL9, CXCL10, CXCL11) and receptors (CXCR3, CXCR6) characterized the Immunoreactive subtype. Recent studies have revealed a significant contribution of tumor stromal genes to stem/serrated/mesenchymal transcriptional subtype in colorectal cancer^18^,^19^. We sought to assess whether a similar interplay between stromal components and molecular characteristics existed in HGS-OvCa. To this end, tumor purity was inferred by the ABSOLUTE algorithm^24^ and the average purity estimates of Mesenchymal and Immunoreactive samples were significantly lower than those of Differentiated and Proliferative samples (Fig. 2A). An alternative approach using the ESTIMATE method^25^ to predict the fraction of stromal and immune cells produced consistent results in the TCGA dataset (Fig. 2B), as well as in Tothill and Crijns cohorts (Supplementary Figure S6). These data suggested that a higher stromal content was associated with the Mesenchymal and Immunoreactive subtypes and might dominate the observed transcriptional traits. To analytically test this hypothesis, we identified signature genes that were upregulated in the Mesenchymal or Immunoreactive subtypes (Supplementary Table S2). Gene Set Enrichment Analysis (GSEA)^26^ indicated that Mesenchymal and Immunoreactive gene signatures were significantly enriched in the microdissected stroma components in comparison to paired tumor tissues^8^ (Fig. 2C). Additionally, we analyzed gene expression profiles of nine pairs of ovarian tumors and matched patient-derived xenografts (PDXs), in which human stromal cells were substituted by mouse cells^27^. Mesenchymal and Immunoreactive gene transcripts were accordingly depleted in PDXs (Fig. 2D). Collectively, these findings demonstrated that tumor-associated stromal content substantially influenced the transcriptional profiles and molecular subtypes of HGS-OvCa.

### Regulatory networks and master regulators of Mesenchymal and Immunoreactive subtypes

**Figure 3:**
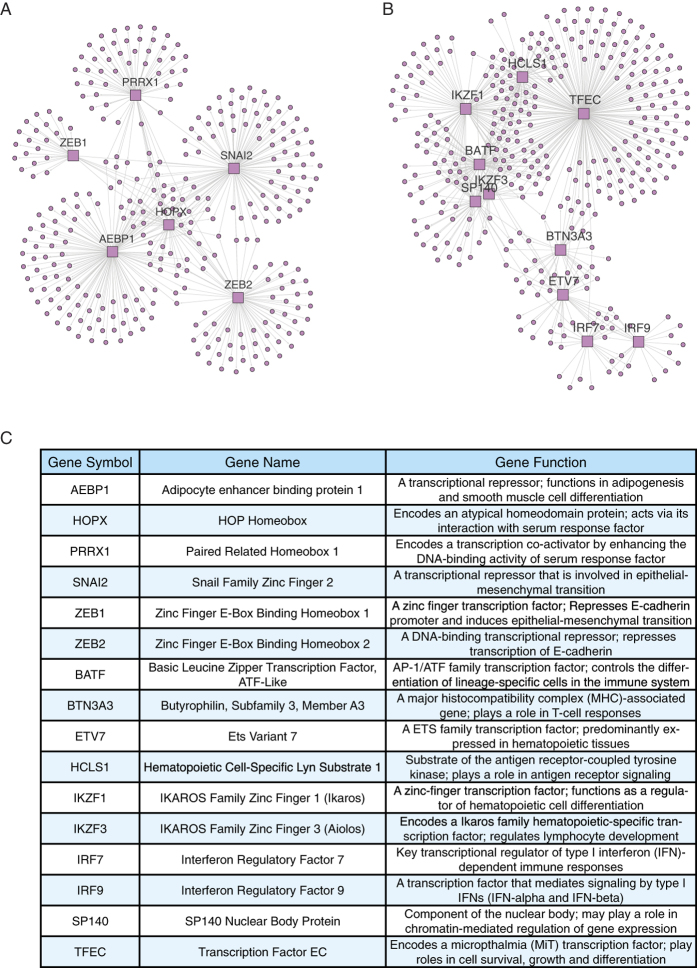
Regulatory networks and master regulators of Mesenchymal and Immunoreactive subtypes. (**A**) The Mesenchymal regulatory network showing the six MRs (square nodes) and all inferred targets (round nodes). (**B**) The Immunoreactive regulatory network showing the ten MRs (square nodes) and all inferred targets (round nodes). (**C**) The list of Mesenchymal and Immunoreactive MRs.

We employed a network-based strategy^28^,^29^,^30^ to uncover the molecular mechanism underlying the discrete HGS-OvCa molecular subtypes, particularly Mesenchymal and Immunoreactive gene programs most correlated with stromal rather than epithelial origin. First, a regulatory network was constructed for ovarian cancer based on the TCGA dataset using a genome-wide reverse engineering approach^28^. Next, we applied the Master Regulator Analysis (MRA) algorithm to the network^29^, to identify regulons showing statistically significant overlap with Mesenchymal or Immunoreactive genes. From a list of 1111 transcription factors (TFs) (Supplementary Table S3), MRA inferred 6 Mesenchymal-specific TFs (Fig. 3A; Supplementary Figure S7; Supplementary Table S4) and 10 Immunoreactive-specific TFs (Fig. 3B; Supplementary Figure S8; Supplementary Table S5), as master regulators (MRs) of HGS-OvCa molecular subtypes. Interestingly, most Mesenchymal MRs had been implicated in epithelial-mesenchymal transition and Immunoreactive MRs were predominantly transcriptional regulators of immune function (Fig. 3C), thus validating the robustness of our approach.

### Mesenchymal and Immunoreactive MRs correlate with tumor stroma and patient survival

**Figure 4:**
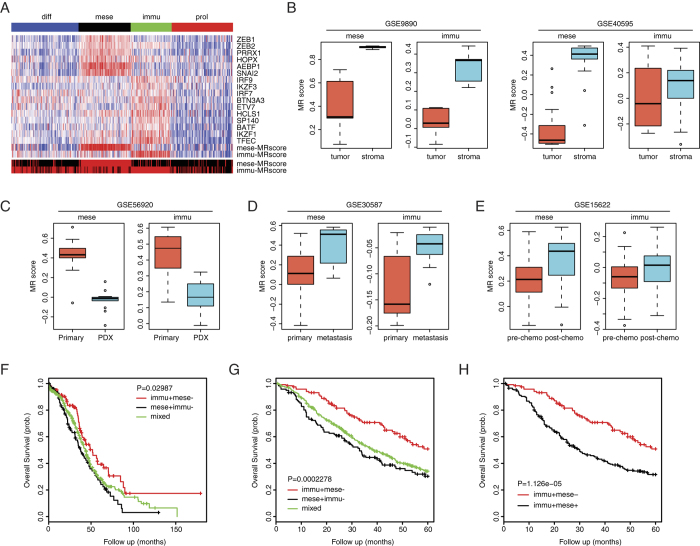
Mesenchymal and Immunoreactive MRs correlate with tumor stroma and patient survival. (**A**) Heatmap of Mesenchymal and Immunoreactive MRs expression and ssGSEA scores for four molecular subtypes of TCGA samples. Binary scores were shown to indicate whether a tumor sample activated Mesenchymal or Immunoreactive MRs. Red, activated; black, not activated. (**B**) Mesenchymal and Immunoreactive MRs scores in microdissected tumor stroma (5 samples in GSE9890 and 31 samples in GSE40595) versus epithelial tissues (5 samples in GSE9890 and 32 samples in GSE40595). (**C**) Mesenchymal and Immunoreactive MRs scores in PDX versus matched primary tumors (9 samples). (**D**) Mesenchymal and Immunoreactive MRs scores in tumor metastasis versus primary tumors (9 paired samples). (**E**) Mesenchymal and Immunoreactive MRs scores in tumors treated with chemotherapy (34 samples) versus non-treated tumors (35 samples). (**F**) Kaplan Meier curves for three prognostic groups of TCGA samples classified by Mesenchymal and Immunoreactive MRs signatures. G. Kaplan Meier curves for meta-analysis of 749 HGS-OvCa expression profiles across five cohorts. H. Kaplan Meier curves for ‘immu + mese−’ and ‘immu + mese+’ patients.

Using MRs as refined physiologically relevant gene signatures, we performed single-sample Gene Set Enrichment Analysis (ssGSEA)^31^ to assess MRs compound scores for the TCGA HGS-OvCa samples. The ssGSEA scores correlated well with those produced by the Gene Set Variation Analysis (GSVA)^32^ as an independent method (Supplementary Figure S9). As expected, the Mesenchymal and Immunoreactive subtypes showed relatively higher levels of Mesenchymal and Immunoreactive MRs expression as well as compound scores, respectively (Fig. 4A). The binary scores indicated that 78% (380/489) of expression profiles showed activity of Mesenchymal or Immunoreactive MRs, and that 28% of tumor samples could be assigned to both Mesenchymal and Immunoreactive subtypes (Fig. 4A), confirming previous observations that HGS-OvCa is highly heterogeneous^11^,^33^. To investigate the relative contributions of epithelial tumor cells or stromal cells to the expression of MRs, we analyzed two gene sets of ovarian tumor samples^8^,^34^, in which epithelial and stromal components had been microdissected and profiled separately. Both Mesenchymal and Immunoreactive MRs were significantly upregulated in tumor stroma in comparison to epithelial tumor areas (Fig. 4B). Consistently, Mesenchymal and Immunoreactive MRs were significantly downregulated in patient-derived xenografts relative to paired primary tumors (Fig. 4C). Therefore, as with HGS-OvCa molecular subtypes, Mesenchymal and Immunoreactive MRs were associated with noncancerous tumor stroma. Further detailed analysis indicated that levels of Mesenchymal and Immunoreactive MRs increased upon metastasis (Fig. 4D) and chemotherapy (Fig. 4E)^35^,^36^, supporting their involvement in tumor progression and response to treatment. Interestingly, the Mesenchymal MRs correlated with gene expression of multiple IGF-related molecules including IGF, IGFBP4, IGFBP6 and IGFBP7, indicative of IGF pathway activation in the Mesenchymal tumors (Supplementary Figure S10).

To assess Mesenchymal and Immunoreactive MRs as prognostic biomarkers, we analyzed their expression in all HGS-OvCa samples included in curatedOvarianData for which overall survival information was available^37^. All 6 Mesenchymal MRs significantly correlated with poor patient outcome (Supplementary Figure S11). Conversely, 7 of 10 Immunoreactive MRs showed significant association with improved overall survival (Supplementary Figure S12). Based on these findings, we subdivided the TCGA samples into three clusters, i.e. ‘immu + mese−’, ‘immu − mese+’ and ‘mixed’, according to Mesenchymal and Immunoreactive compound scores. We found that patients classified as ‘immu − mese+’ had significantly shorter survival than ‘immu + mese−’ patients, and the ‘mixed’ group showed intermediate outcome (Fig. 4F). To independently corroborate the prognostic value of MRs signatures, we performed meta-analysis of 749 HGS-OvCa expression profiles across five datasets^8^,^23^,^38^,^39^,^40^. The difference in survival between the three groups was highly statistically significant (Fig. 4G). Interestingly, compared with ‘immu + mese−’ subtype, patients whose tumor samples express both Mesenchymal and Immunoreactive MRs (‘immu + mese+’) had statistically significantly worse survival (Fig. 4H). On the contrary, we did not observe survival difference between ‘immu − mese+’ and ‘immu + mese+’ groups (Supplementary Figure S13). These data highlighted the dominant role of Mesenchymal MRs in predicting patient outcome.

## Discussion

In this study, we presented a detailed analysis of the four molecular subtypes based on TCGA HGS-OvCa expression data. We showed that Mesenchymal and Immunoreactive subtypes were characterized by transcriptional traits dominated by tumor-infiltrating stromal cells. By systematically interrogating subtype-specific regulatory networks, we further identified the transcriptional module and master regulators that drove the expression of Mesenchymal and Immunoreactive signatures. This approach led to the identification of novel transcription factors as potentially critical regulators of tumor-associated microenvironment, which also served as robust prognostic biomarkers of aggressive ovarian cancer.

We provided several complementary lines of evidence supporting that Mesenchymal and Immunoreactive signature genes were mostly expressed by tumor stromal components. First, tumor purity inferred by the ABSOLUTE or ESTIMATE algorithm was significantly lower in Mesenchymal and Immunoreactive samples than in Differentiated and Proliferative samples, suggesting that a higher stromal content was associated with the Mesenchymal and Immunoreactive subtypes. Second, analysis of the expression profiles of microdissected ovarian cancer demonstrated that Mesenchymal and Immunoreactive gene signatures were significantly enriched in the stroma components in comparison to paired tumor tissues. Third, in nine pairs of ovarian tumors and matched patient-derived xenografts (PDXs), where human stroma was substituted by mouse stroma, Mesenchymal and Immunoreactive gene transcripts were depleted in PDXs. Notably, these findings were confirmed in multiple independent datasets, indicating that abundant stroma was an intrinsic feature of some HGS-OvCa samples rather than a technical artifact.

The four TCGA subtypes, namely Differentiated, Mesenchymal, Immunoreactive and Proliferative, were originally defined by specifically expressed genes in the clusters^10^. However, regulatory mechanisms at the molecular level remain to be discovered within each subtype, in order to elucidate causal drivers and to identify relevant targets for effective cancer treatment. We took a systems biology approach to gain insight into transcriptional networks associated with molecular subtypes and identified central transcription factors as master regulators of Mesenchymal and Immunoreactive phenotypes. Our analysis identified ZEB1, ZEB2, SNAI2, PRRX1, AEBP1 and HOPX as Mesenchymal MRs, most of which had been implicated in epithelial-mesenchymal transition^41^, and IRF7, IRF9, IKZF1, IKZF3, BATF, ETV7, BTN3A3, SP140, HCLS1 and TFEC as Immunoreactive MRs, which were predominantly transcriptional regulators of immune function^42^,^43^,^44^. In line with stromal enrichment of Mesenchymal and Immunoreactive signature genes, Mesenchymal and Immunoreactive MRs were associated with noncancerous tumor stroma. It would be interesting to investigate the biological functions of these MRs in future studies.

Importantly, a statistically significant correlation was observed between the MRs and patient survival. In contrast, the simple classification into four molecular subtypes did not seem to have prognostic relevance. It was previously shown that subtype assignment of HGS-OvCa expression profiles were not mutually exclusive^20^. Therefore, MRs might have increased power to detect Mesenchymal and Immunoreactive traits that were otherwise concealed by sample heterogeneity. Indeed, we found that Mesenchymal or Immunoreactive MRs were detectable in the majority of TCGA samples (78%) at different levels of activation. Furthermore, there is an emerging consensus that network-based biomarker candidates, especially with sound biologic rationale, often exhibit high reproducibility and sensitivity in ovarian cancer^45^,^46^. The facts that stroma-associated MRs predicted clinical outcome and that Mesenchymal and Immunoreactive MRs differentially linked to opposite prognostic categories prompted us to deduce that the stromal composition and interplay might have a significant, if not major, contribution to the HGS-OvCa prognosis. These findings may reconcile controversies on the prognostic importance of HGS-OvCa molecular subtypes and propose a simple way to subgroup patients for accurate management. Further validation of stromal MRs in forecasting disease outcome will require prospective clinical studies, preferably using feasible assays that can process many samples on a routine basis, e.g. quantitative PCR or immunohistochemistry.

In addition to patient prognosis, we also found that Mesenchymal and Immunoreactive MRs were upregulated in tumor metastasis or in response to chemotherapy. Moreover, the Mesenchymal MRs compound scores correlated with expression levels of multiple IGF-related genes, suggesting that the IGF pathway might be a druggable target in ovarian tumors with Mesenchymal MRs signature. Future studies should further elucidate the intricate relationship between tumor subtypes, MRs expression, tumor progression, response to treatment and patient outcome. For example, individuals with Immunoreactive MRs signature may benefit from treatments targeting tumor cells via the immune response, such as recently approved immune checkpoint inhibitors or vaccine therapies in the adjuvant setting. On the other hand, patients expressing Mesenchymal MRs exhibit poor outcome and probably need to be treated more aggressively with chemotherapy. We speculate that beyond disease prognosis, insights into stroma-associated MRs may contribute to the selection and development of new therapeutic strategies.

In summary, integrative analyses presented in this work determined the cellular and molecular underpinnings of HGS-OvCa subtypes. We provided evidence that infiltrating stromal cells had a profound effect on the expression patterns of HGS-OvCa, particularly Mesenchymal and Immunoreactive clusters. A stroma-associated gene signature composed of transcriptional master regulators was inferred by unbiased reverse engineering algorithm, and proved to effectively stratify patients into different prognostic groups. Therefore, systematic interrogation of genome-wide context-specific networks may not only advance our understanding of the regulatory programs underlying cancer phenotypes, but also enable accurate prediction of patient prognosis. Importantly, the MRs signature only consists of 16 genes, making clinical implementation using various gene/protein profiling platforms feasible. We envision that our findings should provide a basis for improved stratification of patients with HGS-OvCa that may ultimately lead to more precise therapies.

## Methods

### Microarray datasets

We used various microarray datasets of HGS-OvCa in the public domain. Combined and filter TCGA gene expression data were downloaded from https://tcga-data.nci.nih.gov/docs/publications/ov_2011/. The five patient cohorts (Tothill, Crijns, Bonome, Yoshihara and Denkert) for meta-analysis have been described previously^8^,^23^,^38^,^39^,^40^, and processed data were downloaded from a recent report^20^. Other microarray datasets, including GSE9890, GSE15622, GSE30587 and GSE56920, are publicly available in NCBI GEO database.

### Identification of molecular subtypes and signature genes

We classified TCGA HGS-OvCa based on non-negative matrix factorization (NMF) consensus clustering originally used to define the four molecular subtypes^10^. NMF is an unsupervised technique to reduce the dimensionality of gene expression data. A small number of metagenes are defined as a positive linear combination of many genes. The metagene expression patterns provide a robust clustering of samples^47^. The NMF classification was confirmed using k-means clustering algorithm implemented in R package ‘ConcensusClusterPlus’^48^. The k-means clustering algorithm aims to classify a given dataset into k clusters, which have been specified a priori. The samples are assigned to the nearest k cluster centroids, and each cluster center is recalculated as the mean value of cluster members, followed by sample reassignment. This process is repeated until the distance between consecutive cluster centers converges. Both the NMF and k-means clustering methods yielded four robust transcriptomic clusters. To minimize the impact of outlier samples on the identification of subtype markers, the silhouette width was computed to filter out expression profiles with negative values, which excluded 101 samples that were not likely a robust representative of the subclass. To identify subtype-specific signature genes, we used significance analysis of microarrays (SAM) to identify genes significantly differentially expressed across the four subtypes. These genes were trained by prediction analysis for microarrays (PAM) to achieve the lowest prediction error, which resulted in the 749-gene signature (Supplementary Table S1). To validate the presence of four molecular subtypes in additional datasets, the 749-gene signature was applied to Tothill and Crijns cohorts, followed by consensus-based NMF analysis. Heatmaps were generated using GenePattern^49^.

### GSEA and ssGSEA

Gene Set Enrichment Analysis (GSEA) was performed as described^26^, using Mesenchymal and Immunoreactive signatures as gene sets. We downloaded the GSEA software from the Broad Institute GSEA portal. Single sample GSEA (ssGSEA) was applied to generate compound scores for Mesenchymal and Immunoreactive master regulators as gene signatures^31^. The procedure was similar to GSEA but gene expression values were ranked for a given sample, and an enrichment score was calculated based on the normalized rank difference in Empirical Cumulative Distribution Functions (ECDF) of the genes in the signature and the remaining genes. We normalized the scores by the absolute difference between the minimum and the maximum for all samples within a dataset before combining ssGSEA scores across different datasets. The Gene Set Variation Analysis (GSVA) was performed to compare the enrichment scores produced by independent methods^32^.

### Tumor purity analysis

For tumor purity analysis, we used two different previously validated approaches, ABSOLUTE and ESTIMATE^24^,^25^. The ABSOLUTE method predicts tumor purity based on the allelic copy-ratio profiles derived from SNP arrays. The ESTIMATE analyses quantify non-tumor constituents by identifying specific gene signatures related to the infiltration of normal cells in tumor tissues. Tumor purity inferred by the ABSOLUTE algorithm was obtained from the TCGA working group. ESTIMATE scores, which predict the level of infiltrating non-tumor cells, were calculated by performing ssGSEA as reported. We first defined stromal and immune scores based on the genes related to stromal tissue and immune cell infiltration, and then combined the stromal and immune scores as the ESTIMATE scores.

### Transcriptional network inference and master regulator analysis

Combined and filter TCGA HGS-OvCa gene expression data were used to build a gene regulatory network. The analysis was conducted with Bioconductor package ‘RTN’^30^, which re-implemented ARACNe in R for reconstruction and analysis of transcriptional networks using mutual information (MI). Transcriptional regulatory units, termed regulons, were assembled by computing the MI between transcription factors (TF) and all potential targets using gene expression data, followed by multiple hypothesis testing corrections (Benjarnini-Hochberg). The TF list was derived from a previous publication^50^. Unstable TF-gene interactions were removed by bootstrap analysis, and Data Processing Inequality (DPI) algorithm was used to remove redundant interactions and preserve the dominant TF-gene pairs. In order to identify subtype-specific transcription factors, the Master Regulator Analysis (MRA) pipeline was applied to estimate the statistical significance of the overlap between the regulons and Mesenchymal or Immunoreactive signature genes using the hypergeometric distribution. GSEA was performed to validate the MRA results. For network visualization we used the Bioconductor package ‘RedeR’^51^.

### Survival analysis

To test the individual Mesenchymal and Immunoreactive master regulators as prognostic markers, we analyzed the hazard ratio of MRs expression and generated forest plots using the ‘curatedOvarianData’ Bioconductor package^37^. For meta-analysis of 749 HGS-OvCa expression profiles, ssGSEA compound scores of Mesenchymal and Immunoreactive master regulators were computed for each sample. The patients were dichotomized into a high-score and a low-score group, using the median ssGSEA score as the threshold value. Based on both Mesenchymal and Immunoreactive compound scores, we stratified samples into three clusters, i.e. ‘immu + mese−’, ‘immu − mese+’ and ‘mixed’. Overall survival curves were calculated using the Kaplan–Meier method, and statistical significance was assessed using the log-rank test. The analyses were conducted with the R Bioconductor ‘survival’ package.

## Additional Information

**How to cite this article**: Zhang, S. *et al.* Stroma-associated master regulators of molecular subtypes predict patient prognosis in ovarian cancer. *Sci. Rep.* **5**, 16066; doi: 10.1038/srep16066 (2015).

## Supplementary Material

Supplementary Information
