# Supplementary material for: Stroma-associated master regulators of molecular subtypes predict patient prognosis in ovarian cancer
Source: Sci Rep. 2015 Nov 4;5:16066. doi: 10.1038/srep16066 (PMC4632004; doi:10.1038/srep16066)
Supplement: Supplementary Information [file srep16066-s1.pdf]

SUPPLEMENTARY INFORMATION

Stroma-associated master regulators of molecular subtypes predict patient prognosis in ovarian cancer

Shengzhe Zhang[1,2,#], Ying Jing[1,#], Meiying Zhang[3,4,#], Zhenfeng Zhang[1,5], Pengfei Ma[1], Huixin Peng[1], Kaixuan Shi[2], Wei-Qiang Gao[1,2,*], Guanglei Zhuang[1,*]

[1]State Key Laboratory of Oncogenes and Related Genes, Renji-Med X Clinical Stem Cell Research Center, Ren Ji Hospital, School of Medicine, Shanghai Jiao Tong University, Shanghai, China
[2]School of Biomedical Engineering & Med-X Research Institute, Shanghai Jiao Tong University, Shanghai, China
[3]Department of Obstetrics and Gynecology, Ren Ji Hospital, School of Medicine, Shanghai Jiao Tong University, Shanghai, China
[4]Shanghai Key Laboratory of Gynecologic Oncology, Ren Ji Hospital, School of Medicine, Shanghai Jiao Tong University, Shanghai, China
[5]State Key Laboratory of Oncogenes and Related Genes, Shanghai Cancer Institute, Ren Ji Hospital, School of Medicine, Shanghai Jiao Tong University, Shanghai, China

[*]Corresponding author:
Guanglei Zhuang
Email: zhuangguanglei@gmail.com
or
Wei-Qiang Gao
Email: gao.weiqiang@sjtu.edu.cn

[#]These authors contributed equally to this work.

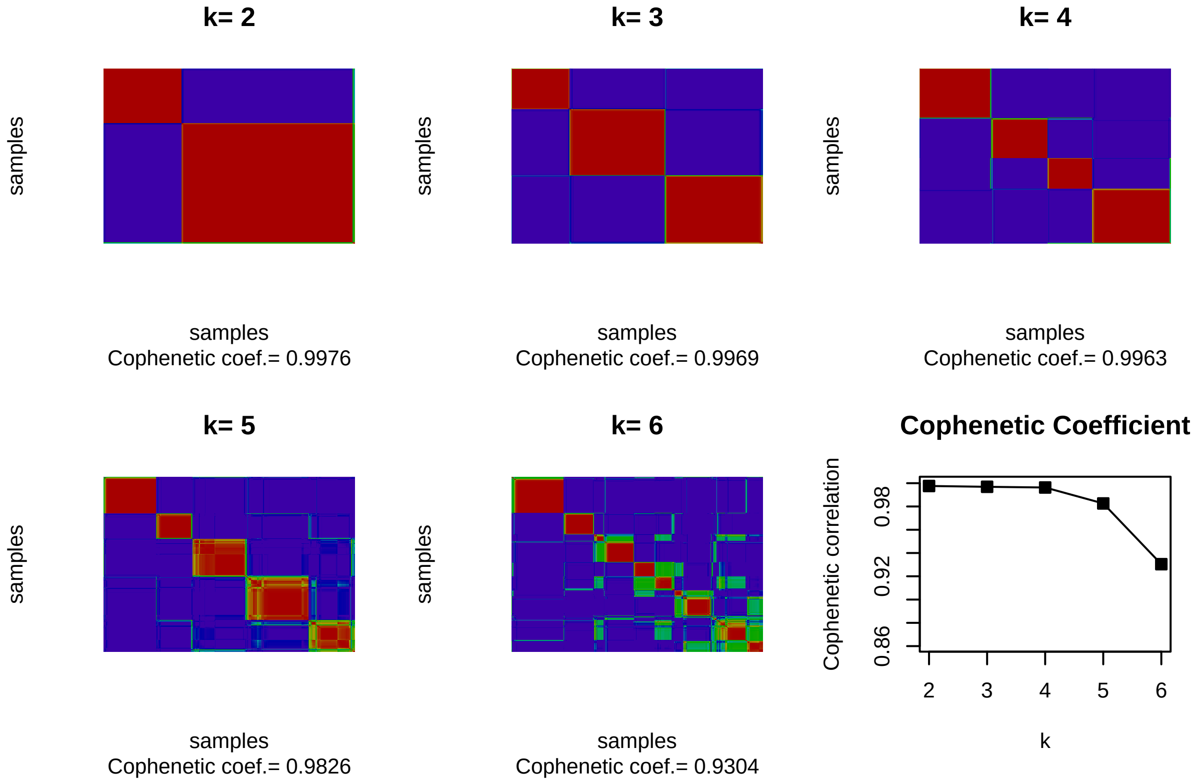

Supplementary Figure 1. NMF consensus clustering analysis and cophenetic coefficient for cluster k=2 to k=5 of the TCGA dataset.

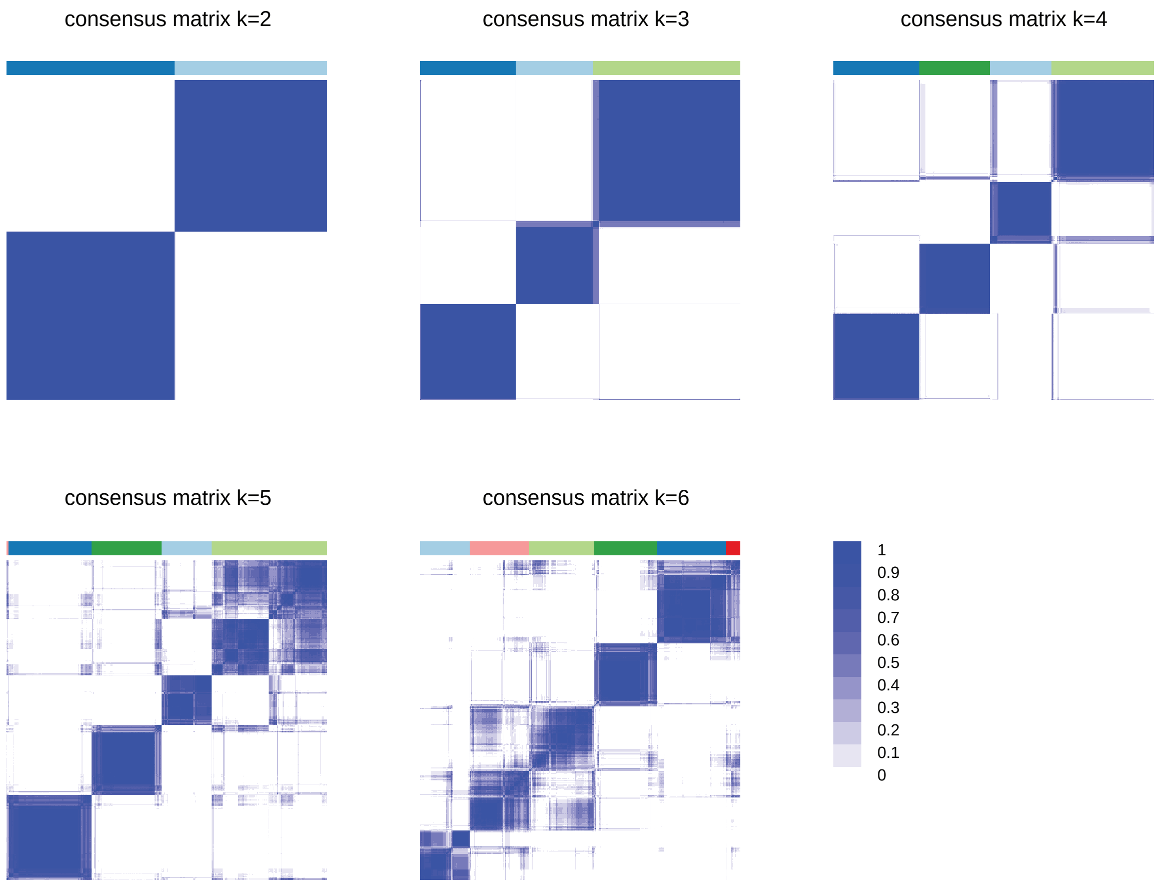

Supplementary Figure 2. k-means consensus clustering analysis of the TCGA dataset.

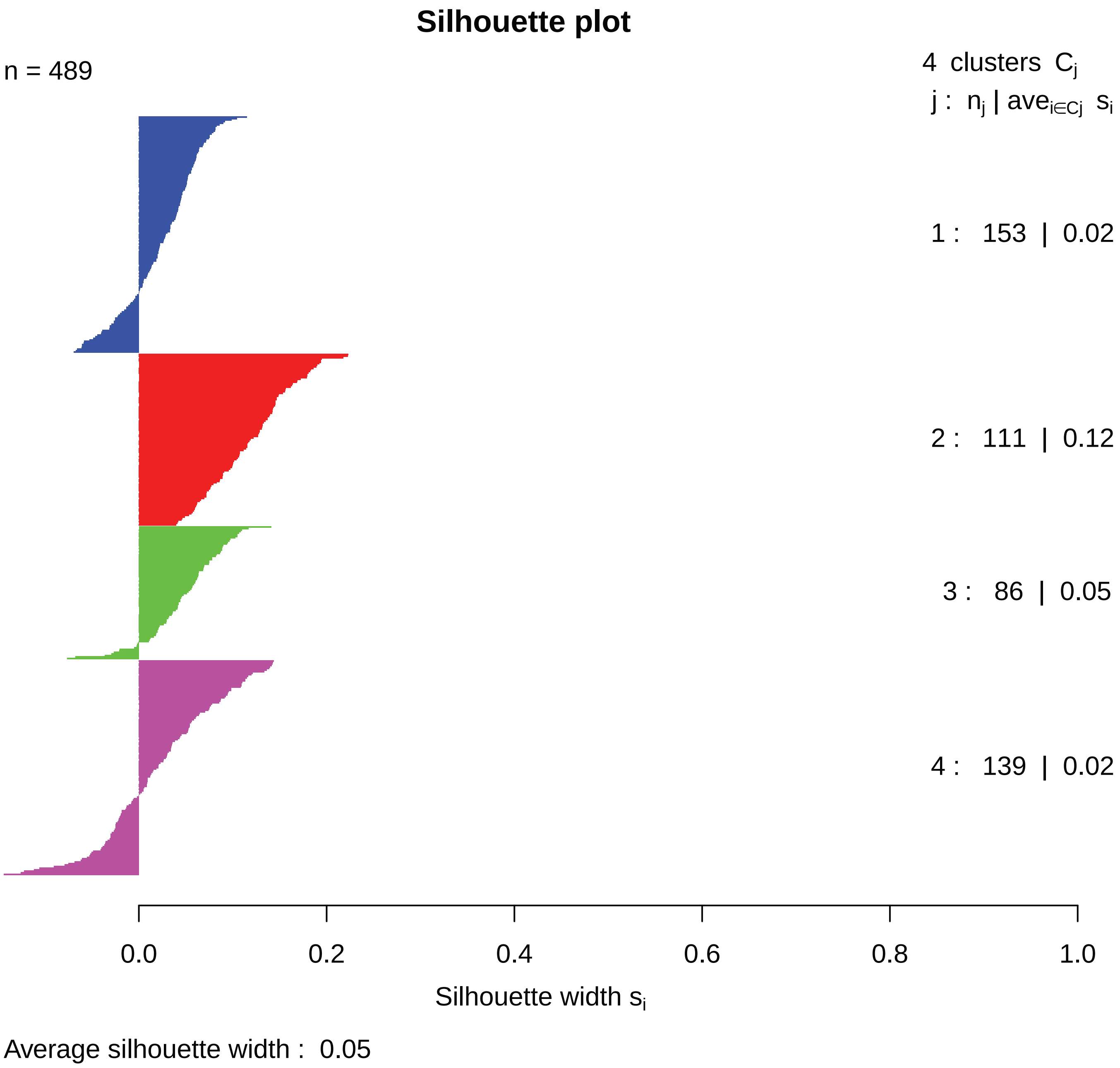

Supplementary Figure 3. Silhouette plot for the TCGA dataset showing samples from different subtypes with positive and negative silhouette score.

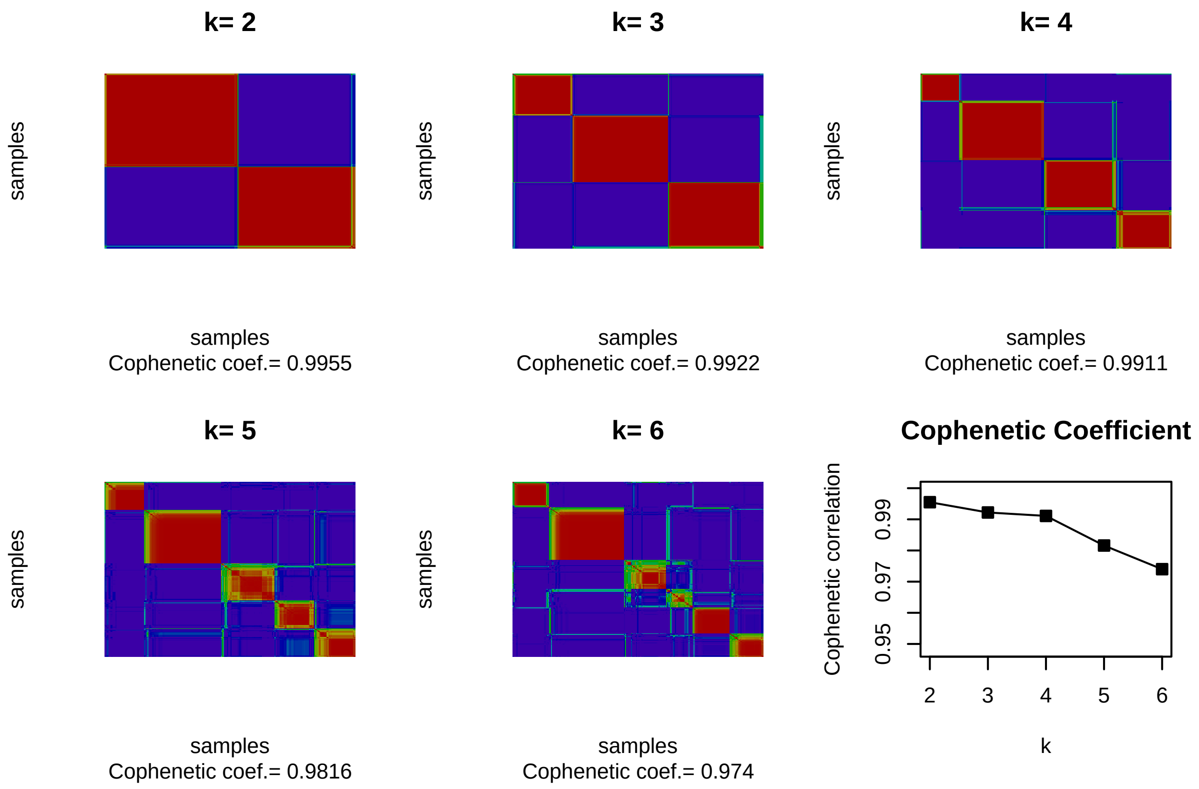

Supplementary Figure 4. NMF consensus clustering analysis and cophenetic coefficient for cluster k=2 to k=5 of the Tothill dataset.

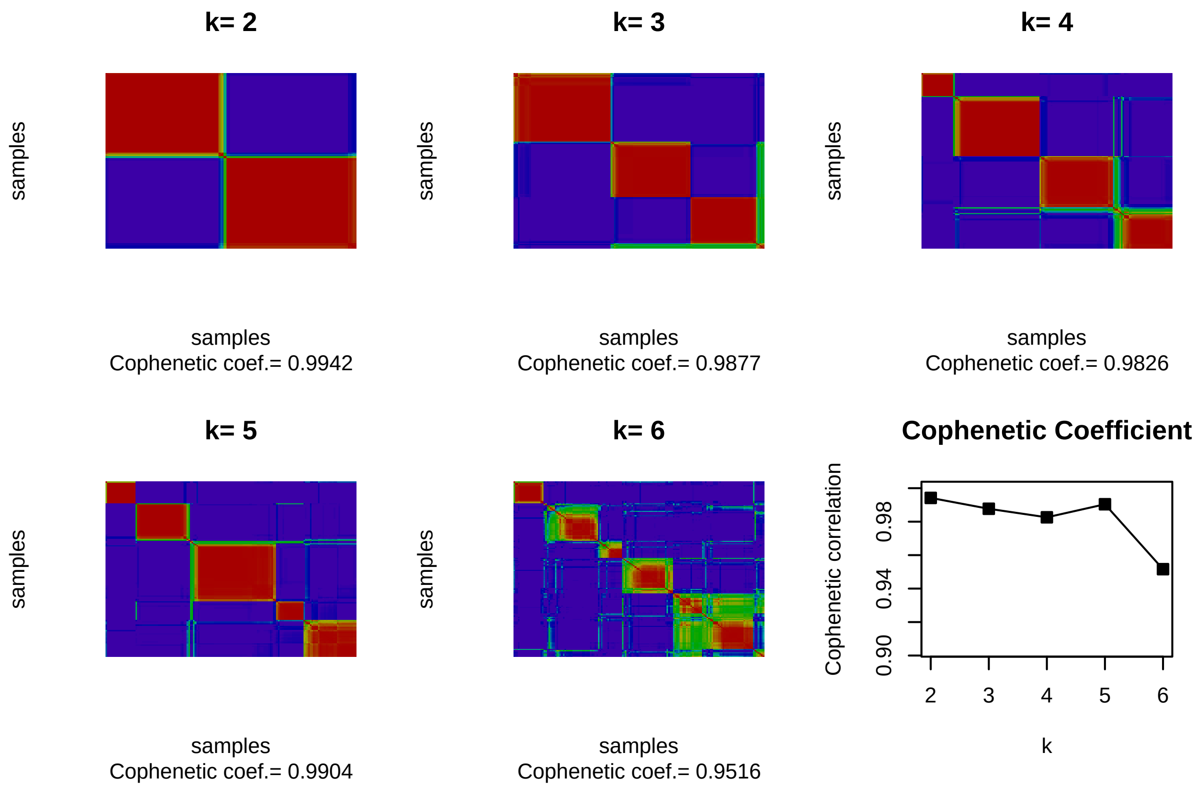

Supplementary Figure 5. NMF consensus clustering analysis and cophenetic coefficient for cluster k=2 to k=5 of the Crijns dataset.

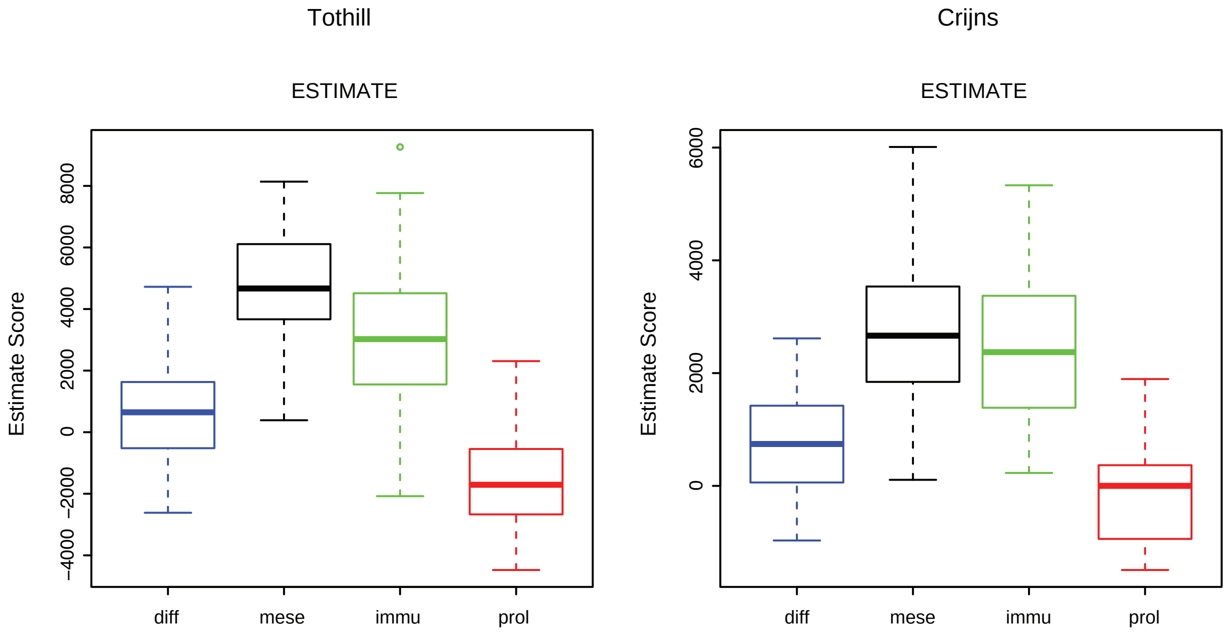

Supplementary Figure 6. ESTIMATE scores for four molecular subtypes of Tothill and Crijns datasets.

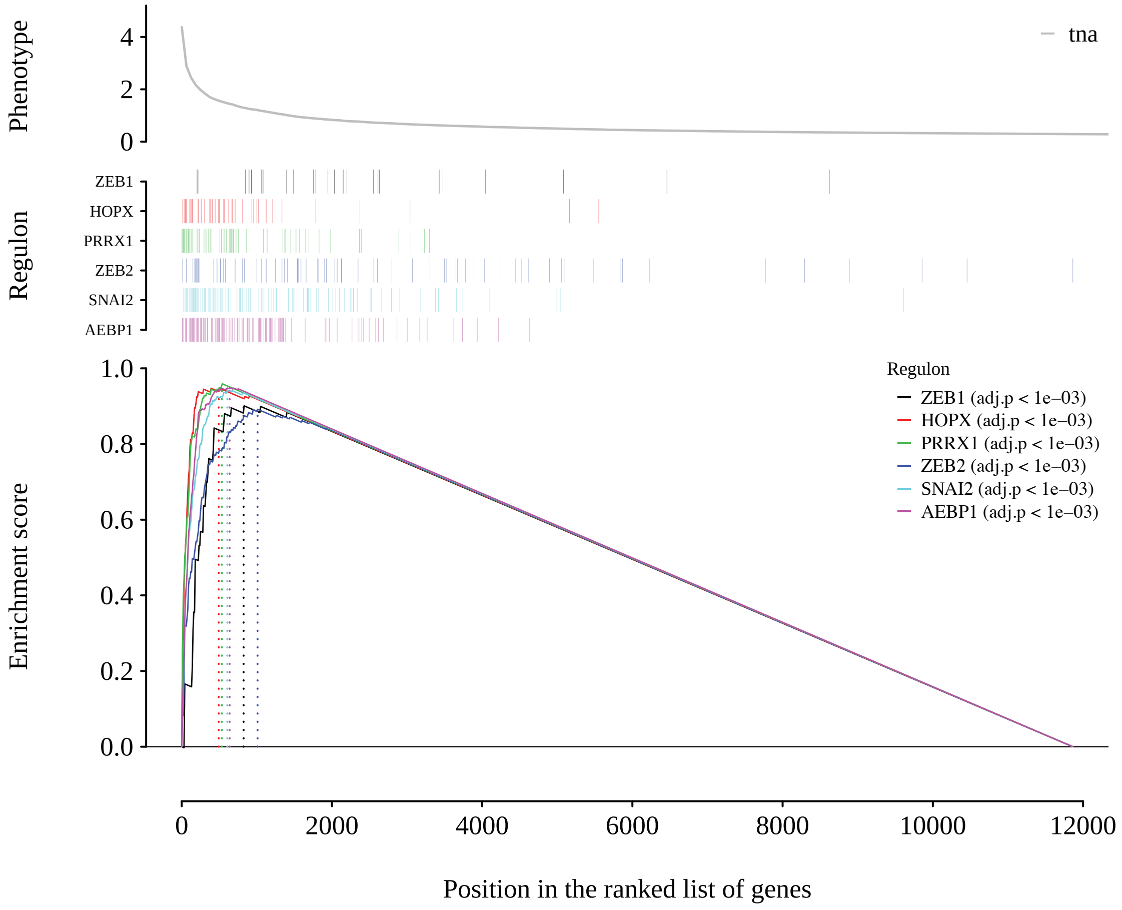

Supplementary Figure 7. GSEA of the genes in Mesenchymal MR regulons.

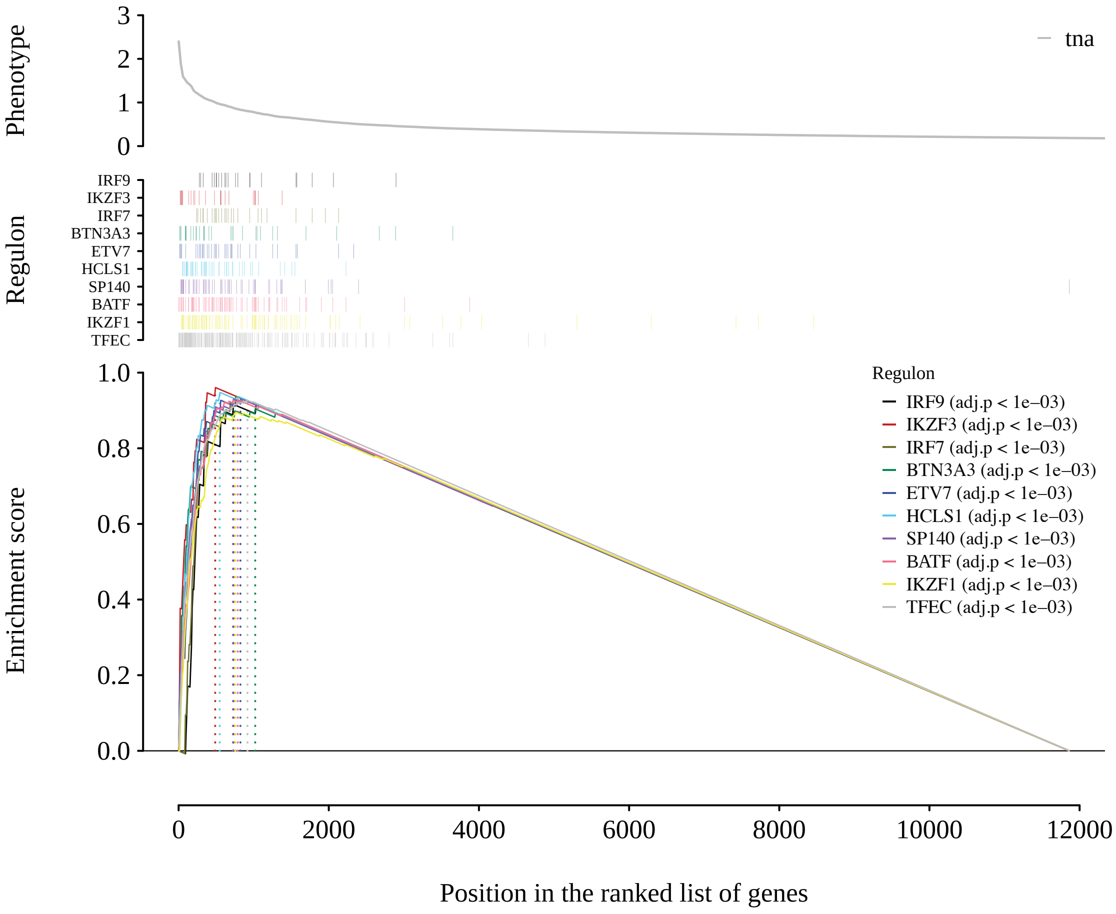

Supplementary Figure 8. GSEA of the genes in Immunoreactive MR regulons.

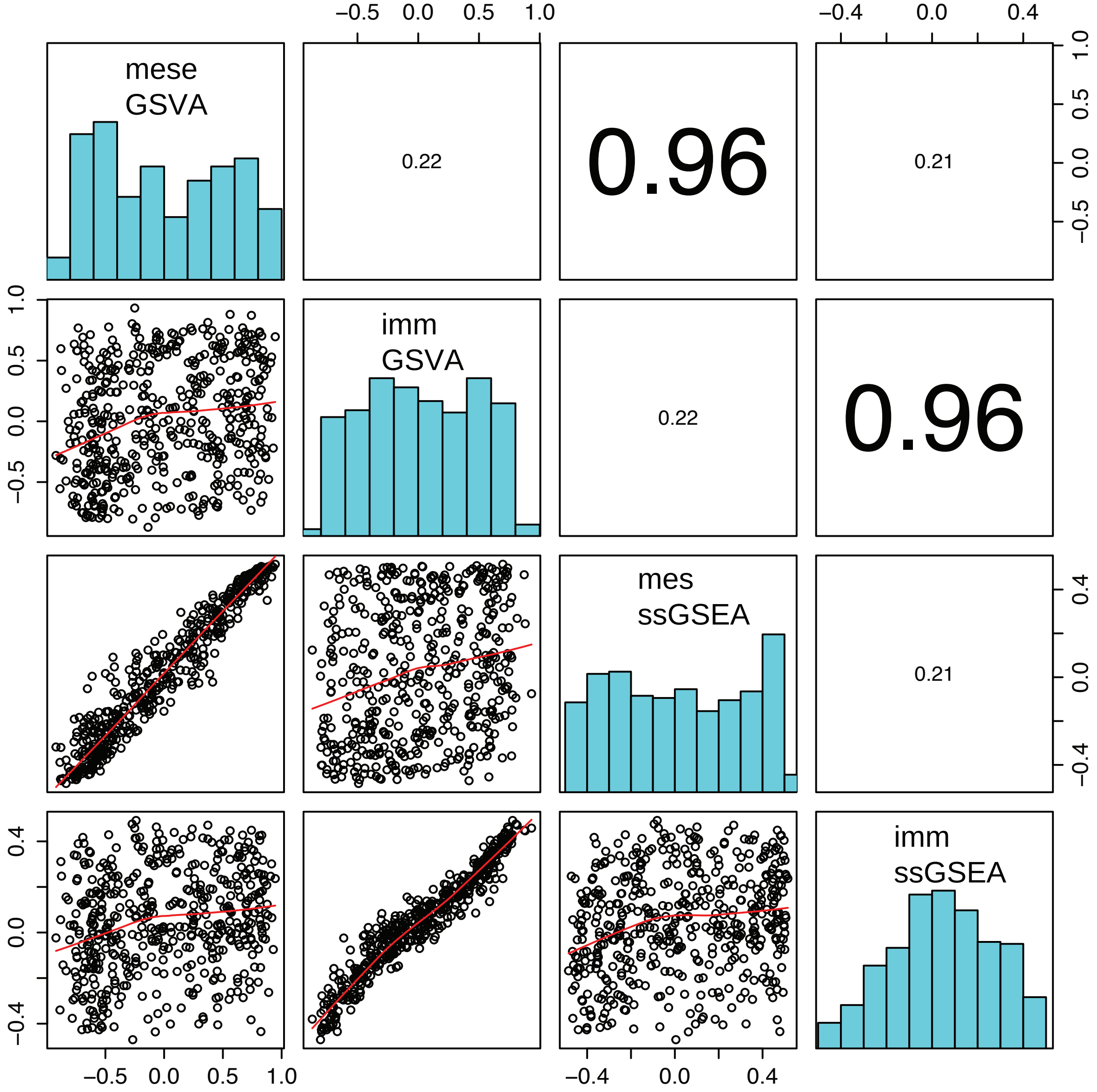

Supplementary Figure 9. Correlation between MRs compound scores for the TCGA HGS-OvCa samples produced by ssGSEA or GSVA.

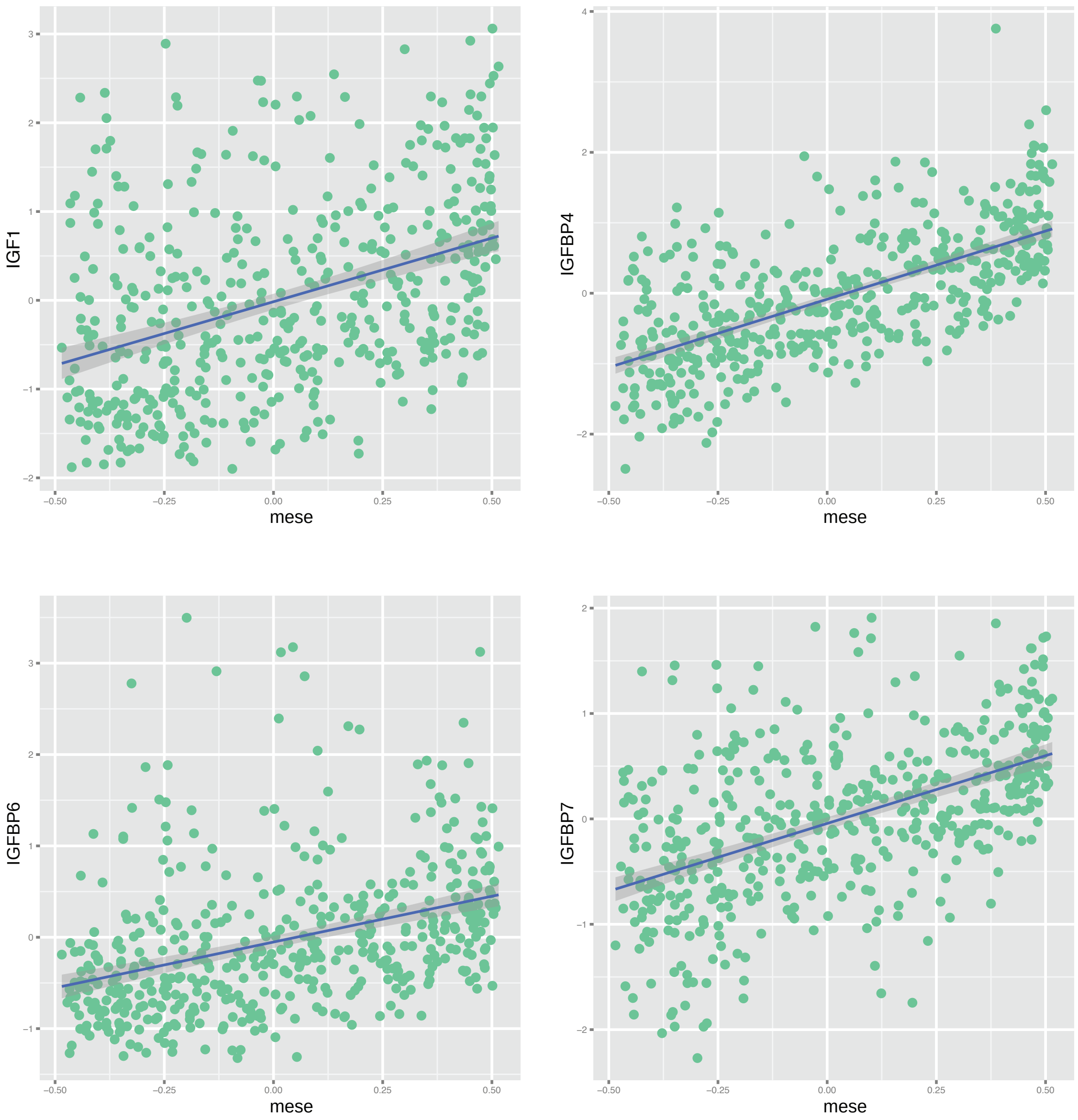

Supplementary Figure 10. Correlation between Mesenchymal MRs and IGF-related genes.

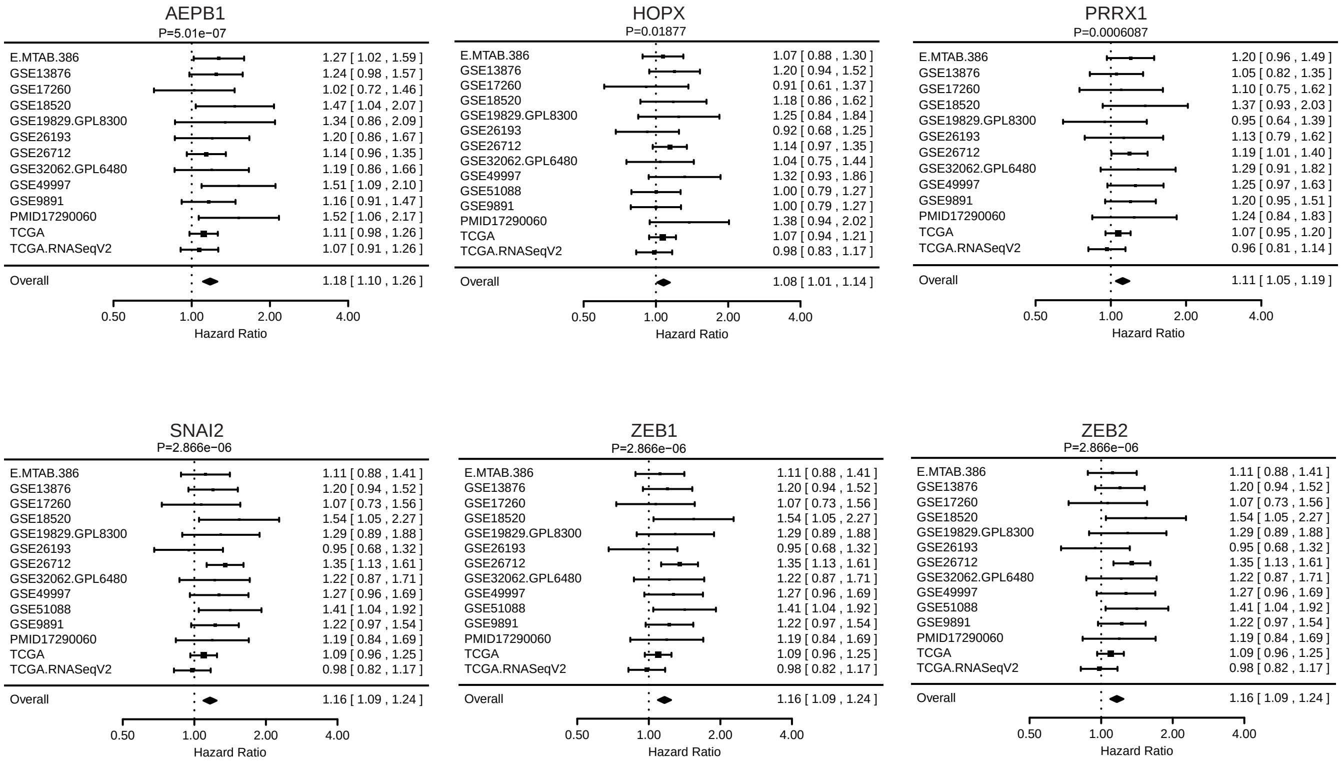

Supplementary Figure 11. Forest plot of Mesenchymal MRs expression as a univariate predictor of overall survival, using HGS-OvCa datasets with applicable expression and survival information.

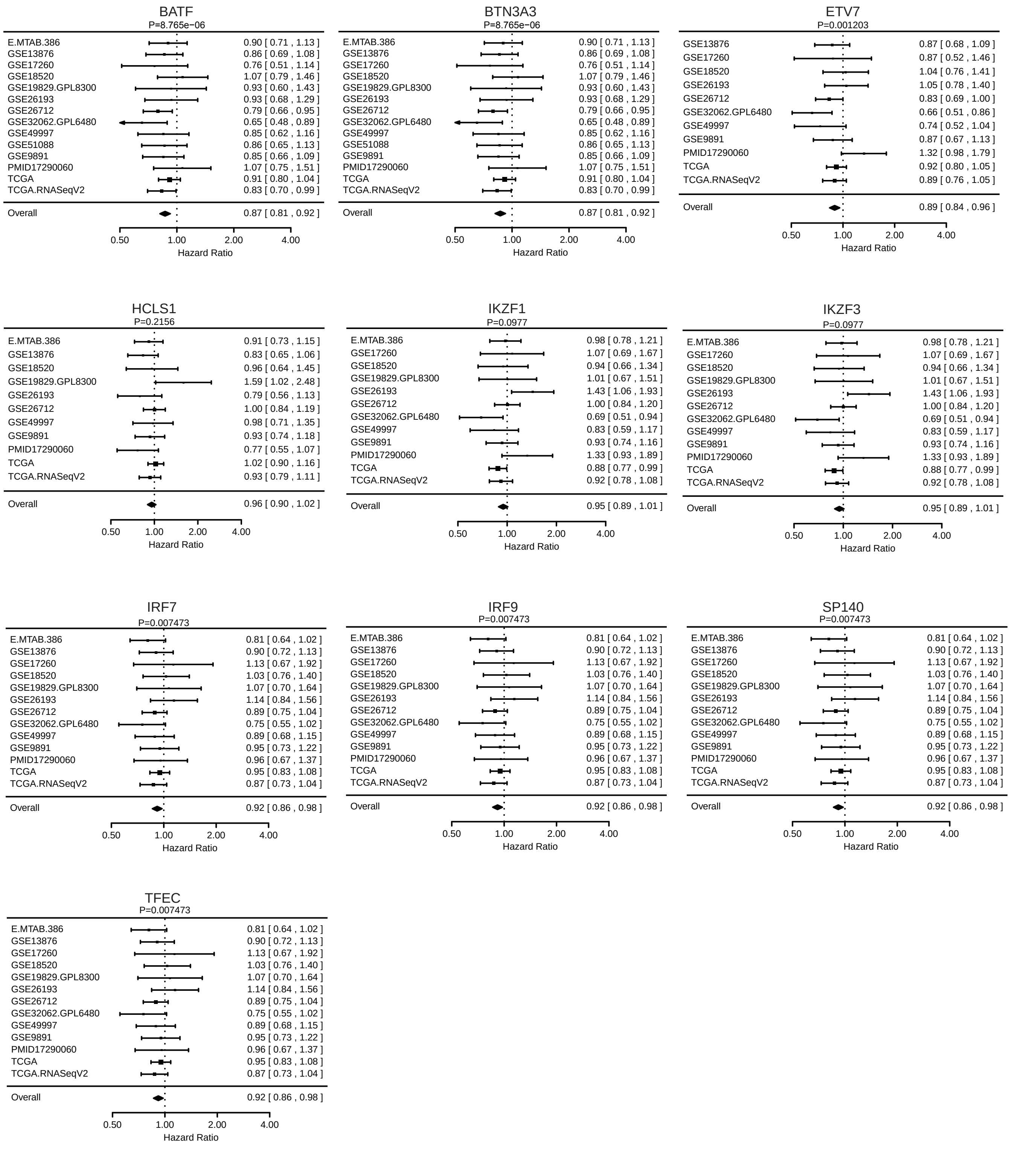

Supplementary Figure 12. Forest plot of Immunoreactive MRs expression as a univariate predictor of overall survival, using HGS-OvCa datasets with applicable expression and survival information.

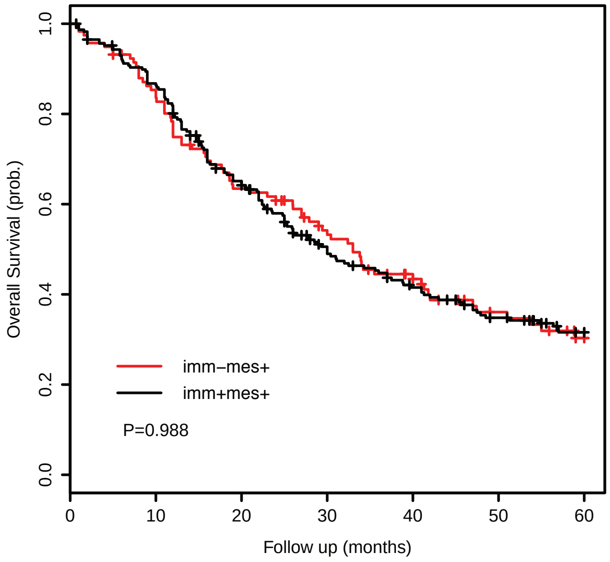

Supplementary Figure 13. Kaplan Meier curves for 'immu-mese+' and 'immu+mese+' patients.

Supplementary Table S1
749-gene classifier with the lowest prediction error

ID	Diff-score	Mese-score	Immu-score	Prol-score
COL5A2	-0.5936	1.3	-0.3419	-0.5793
THBS2	-0.5485	1.2882	-0.1969	-0.7488
FAP	-0.596	1.2865	-0.1388	-0.734
POSTN	-0.6145	1.2394	-0.0612	-0.7164
VCAN	-0.5769	1.1819	-0.2429	-0.536
CTSK	-0.5619	1.1736	-0.0437	-0.7168
INHBA	-0.5077	1.1492	-0.2818	-0.5522
COL10A1	-0.4637	1.1385	-0.2835	-0.5952
ASPN	-0.5157	1.0883	-0.3101	-0.4396
NNMT	0.0332	0.7185	0.145	-1.0855
LUM	-0.544	1.0791	-0.1154	-0.5582
LRRC15	-0.4125	1.0639	-0.362	-0.5001
COL5A1	-0.4549	1.0462	-0.4167	-0.3743
FBN1	-0.4912	1.035	-0.3351	-0.3822
MMP2	-0.4416	1.0315	-0.3448	-0.4351
CD74	0.1971	0.1408	0.6789	-1.0255
COL1A1	-0.4179	0.9972	-0.366	-0.4044
AEBP1	-0.4676	0.9958	-0.3962	-0.3108
HLA-DPA1	0.0512	0.2223	0.7389	-0.9884
EPYC	-0.4519	0.9874	-0.1784	-0.5086
COL3A1	-0.4814	0.9758	-0.2761	-0.3706
CRISPLD2	-0.4531	0.9723	-0.337	-0.3511
GBP2	0.1528	0.2532	0.5147	-0.9688
HLA-DRA	0.0857	0.1871	0.7123	-0.9662
C3	0.2852	0.266	0.281	-0.9586
HLA-DPB1	0.0079	0.2461	0.7354	-0.9584
SNAI2	-0.4686	0.9539	-0.2683	-0.3663
SERPINF1	-0.5204	0.9503	-0.0403	-0.4899
COL6A3	-0.3865	0.9451	-0.3055	-0.4316
CD53	-0.235	0.4052	0.854	-0.9425
PLAU	-0.363	0.9379	0.0304	-0.743
CDH11	-0.4101	0.93	-0.3668	-0.3283
LAPTM5	-0.1962	0.4126	0.7686	-0.9296
DCN	-0.4378	0.9273	-0.1731	-0.4552
RARRES3	0.4096	-0.088	0.5609	-0.9127
FN1	-0.4824	0.9124	0.0021	-0.5282
CXCL9	-0.3746	0.2355	0.9074	-0.5875
COL1A2	-0.364	0.9059	-0.3851	-0.3426
HLA-DMA	0.1408	0.0867	0.7048	-0.9042
CYBB	-0.1732	0.3488	0.792	-0.8988

CASP1	0.0634	0.1689	0.695	-0.8985
CTSS	-0.0457	0.2279	0.7677	-0.8921
SPARC	-0.4662	0.8895	-0.3335	-0.2311
ADAMDEC1	-0.3701	0.2181	0.8883	-0.5549
COLEC12	-0.4221	0.8879	-0.1507	-0.445
HNT	-0.3636	0.8836	-0.2335	-0.4454
SFRP4	-0.3726	0.8811	-0.1273	-0.5218
SQRDL	0.2168	0.091	0.5543	-0.8805
ADAMTS12	-0.4172	0.8777	-0.3261	-0.2871
PTPRC	-0.2597	0.4245	0.7864	-0.8761
AIM2	-0.236	0.0739	0.8753	-0.5368
ITGB2	-0.1021	0.374	0.6168	-0.8741
CD2	-0.2878	0.2724	0.8726	-0.7194
EVI2B	-0.2142	0.4029	0.7408	-0.8696
CD38	-0.1901	-0.0494	0.8677	-0.4338
CXCL11	-0.1007	0.0847	0.865	-0.7206
CCL5	-0.1995	0.1791	0.8621	-0.708
COPZ2	-0.4221	0.8588	-0.1867	-0.3768
CD48	-0.2702	0.258	0.8486	-0.7037
VCAM1	-0.4198	0.8483	0.2401	-0.7343
UBD	-0.1794	0.0186	0.8454	-0.5153
RGS1	-0.1123	0.4297	0.5098	-0.8393
GLT8D2	-0.4193	0.8391	-0.4058	-0.1665
CXCL14	-0.429	0.835	-0.2482	-0.2843
FCER1G	-0.2145	0.3526	0.7733	-0.833
TCF7L1	-0.3193	0.0221	-0.5074	0.8314
THBS1	-0.2842	0.8308	-0.2318	-0.4845
TYROBP	-0.1718	0.3196	0.7531	-0.8299
EVI2A	-0.3042	0.5842	0.564	-0.8294
LCN2	0.4767	-0.0842	0.3513	-0.8256
LYZ	-0.3658	0.3918	0.8255	-0.7281
ALOX5AP	-0.1241	0.3335	0.6541	-0.8253
ECM1	-0.3729	0.8232	-0.1648	-0.4152
OLFML2B	-0.4385	0.821	-0.0583	-0.4177
ACTA2	-0.3712	0.8193	-0.2053	-0.3777
CXCL13	-0.3521	0.1815	0.8184	-0.4717
PSMB9	0.0844	-0.028	0.8137	-0.7773
IL2RG	-0.2178	0.1782	0.8136	-0.6408
TIMP3	-0.4143	0.8116	-0.4083	-0.136
ACTG2	-0.3986	0.8115	-0.234	-0.3067
COL8A1	-0.2957	0.8102	-0.2606	-0.4183
TNFAIP6	-0.494	0.8093	0.1722	-0.528
DPYD	-0.0528	0.3371	0.5192	-0.8079
PDLIM3	-0.344	0.8074	-0.2633	-0.3485
MS4A6A	-0.2218	0.3768	0.718	-0.8065
TFEC	-0.2272	0.2121	0.8029	-0.6625

CD163	-0.2195	0.4477	0.6044	-0.8021
APOL6	0.1203	0.1194	0.5683	-0.8012
TDO2	-0.4273	0.7997	0.0944	-0.5369
TMSL8	-0.3979	-0.0449	-0.2485	0.7976
GPNMB	-0.2345	0.5904	0.4108	-0.7975
LCP2	-0.1849	0.3092	0.7488	-0.7957
C1QA	-0.2467	0.3443	0.7896	-0.7939
ITGBL1	-0.312	0.7913	-0.1979	-0.4265
CD14	-0.1456	0.3924	0.5601	-0.7911
GIMAP4	-0.2209	0.339	0.7531	-0.7898
C3AR1	-0.2055	0.3963	0.6408	-0.7865
INDO	0.1566	-0.0035	0.6769	-0.786
TMEM158	-0.2927	0.7837	-0.2301	-0.4147
ARHGDIB	-0.0588	0.3166	0.5249	-0.7787
CLEC4E	-0.2425	0.1265	0.7773	-0.5109
COL6A2	-0.3061	0.7694	-0.3374	-0.2861
HEPH	-0.2848	0.7656	-0.2711	-0.3667
CXCL12	-0.5271	0.7613	-0.0244	-0.2536
ECM2	-0.2703	0.7611	-0.3222	-0.336
SAMSN1	-0.248	0.3447	0.7488	-0.7575
CCL11	-0.3859	0.7572	0.1087	-0.5497
EDIL3	-0.3228	0.7556	-0.3475	-0.2378
FMO1	-0.3035	0.7549	-0.1861	-0.4015
MNDA	-0.2131	0.3569	0.6702	-0.7514
NUAK1	-0.4963	0.7502	-0.331	-0.0157
APOL3	0.0952	0.0814	0.6025	-0.749
SERPINE1	-0.3235	0.7489	-0.1891	-0.3647
AIF1	-0.1643	0.3221	0.6433	-0.7484
C1QB	-0.2609	0.3499	0.7484	-0.7468
RNASE6	-0.1666	0.2877	0.6977	-0.7483
IRF1	0.1836	-0.0171	0.6059	-0.7431
FCGR2A	-0.2063	0.4216	0.5537	-0.7426
PCOLCE	-0.4101	0.7422	-0.335	-0.116
GPR65	-0.1913	0.2648	0.7403	-0.7232
CLEC2B	-0.2537	0.6585	0.2719	-0.7392
C1QTNF3	-0.3465	0.7385	-0.4002	-0.1392
RAB31	-0.3618	0.7373	0.1171	-0.5634
DACH1	-0.2265	-0.06	-0.4167	0.7351
TAP1	0.0491	-0.0425	0.7351	-0.6443
TGFBI	-0.3144	0.7349	0.2285	-0.719
APOL1	0.0916	0.1894	0.4316	-0.7348
TRIM22	-0.0772	0.2691	0.5724	-0.7347
COMP	-0.4423	0.7343	-0.2393	-0.1459
IL7R	-0.365	0.6737	0.4133	-0.7333
APBB1IP	-0.1006	0.2734	0.5979	-0.7313
PLEK	-0.2238	0.3253	0.7076	-0.7292

BGN	-0.3403	0.7256	-0.3437	-0.1797
SCG2	-0.3205	0.7247	-0.1766	-0.3487
FCGR2B	-0.2481	0.3555	0.6939	-0.7238
PDPN	-0.4578	0.7227	-0.2613	-0.0916
MS4A4A	-0.3142	0.4371	0.6727	-0.7222
SULF1	-0.2586	0.7174	0.1371	-0.6917
CD3D	-0.2161	0.1577	0.7116	-0.5289
FGL2	-0.1987	0.2724	0.7097	-0.6966
FYB	-0.1767	0.3106	0.6337	-0.709
VSIG4	-0.25	0.4019	0.6092	-0.7075
EDNRA	-0.2984	0.7042	-0.1934	-0.3372
EBI2	-0.2169	0.4457	0.4893	-0.7037
PSMB10	0.1826	0.0111	0.5179	-0.702
SRGN	-0.2687	0.3521	0.7017	-0.699
IFI30	-0.1472	0.2177	0.6998	-0.6864
NKG7	-0.2069	0.1427	0.6991	-0.5111
APOC1	-0.2482	0.276	0.6981	-0.6259
GMFG	-0.2627	0.369	0.6627	-0.6949
SPON2	-0.3364	0.6949	-0.2813	-0.1993
HMGA2	-0.2473	-0.0438	-0.3584	0.6916
SLAMF8	-0.2921	0.2719	0.6913	-0.5567
VTCN1	0.2808	-0.0027	0.375	-0.6911
GZMH	-0.2117	0.0968	0.689	-0.4375
IL15	0.0811	0.0596	0.5864	-0.6888
TMEFF1	-0.37	0.0141	-0.2497	0.6864
C2	0.109	0.0813	0.5041	-0.6823
LY86	-0.1599	0.2971	0.5951	-0.6806
CD69	-0.1537	0.2824	0.6067	-0.6802
TLR2	-0.1611	0.2866	0.6091	-0.6778
IL10RA	-0.1852	0.2947	0.6341	-0.6777
TNFSF10	0.1394	0.0378	0.5156	-0.677
SRPX	-0.4028	0.6758	-0.3919	0.008
GFPT2	-0.2063	0.6729	-0.2057	-0.4085
SRPX2	-0.2258	0.6728	-0.2933	-0.307
CD52	-0.2503	0.2376	0.6721	-0.5516
PSCDBP	-0.333	0.348	0.6718	-0.583
HLA-E	0.0154	0.1038	0.5959	-0.6665
SEMA3D	-0.4756	0.6649	-0.2579	0.0027
SERPINA1	0.1138	0.1606	0.3582	-0.6641
CXORF21	-0.1788	0.3287	0.5579	-0.664
HLA-B	-0.0144	0.1223	0.6105	-0.6632
CD86	-0.1745	0.2254	0.6599	-0.6259
CCL2	-0.1101	0.341	0.4291	-0.6593
RTP4	0.2701	-0.1112	0.5125	-0.657
IGKC	-0.4495	0.3521	0.6564	-0.421
BIRC3	0.1871	0.0347	0.4206	-0.6543

ALDH1A3	-0.2281	0.6541	-0.1634	-0.3921
CFB	0.2536	-0.0292	0.4127	-0.6538
FXYD6	-0.2409	-0.0377	-0.3267	0.6482
LOXL1	-0.3606	0.6455	-0.3652	-0.0321
TAGLN	-0.2521	0.6455	-0.2498	-0.275
CLEC7A	-0.1602	0.2699	0.5946	-0.6452
CCR5	-0.1711	0.1969	0.6412	-0.5778
NCF2	-0.1997	0.2977	0.6072	-0.6394
HCK	-0.2464	0.2869	0.6387	-0.591
GZMB	-0.1658	0.1119	0.6383	-0.4739
DACT1	-0.3078	0.6368	-0.3515	-0.1026
NT5E	-0.103	0.6364	-0.1674	-0.5316
LAIR1	-0.1998	0.3058	0.5919	-0.6362
ZNF423	-0.1762	-0.0795	-0.3483	0.6346
IL15RA	0.1042	0.0385	0.5161	-0.6317
BEX1	-0.2369	-0.087	-0.2388	0.6299
GEM	-0.3339	0.6297	-0.1452	-0.2369
ARHGAP15	-0.1942	0.2632	0.6295	-0.6218
RCN3	-0.3391	0.6279	-0.2202	-0.1631
SPP1	-0.1359	0.3714	0.3868	-0.6276
HLA-C	0.007	0.102	0.5661	-0.6274
CSF1R	-0.1175	0.2968	0.4685	-0.6272
GBP1	-0.0111	0.1179	0.5691	-0.6264
SLCO2B1	-0.1541	0.333	0.4699	-0.6263
OLFML1	-0.2841	0.6263	-0.1973	-0.2534
HLA-DMB	-0.0261	0.0456	0.6255	-0.5629
IGSF6	-0.1872	0.2407	0.6242	-0.5977
IL2RB	-0.1365	0.1537	0.6237	-0.5533
NBL1	-0.1551	0.6235	-0.2435	-0.3806
IFI16	0.1858	0.0525	0.3598	-0.6228
PMP22	-0.3382	0.6208	-0.1364	-0.2274
CORO1A	-0.0283	0.1508	0.54	-0.6206
APOBEC3G	-0.0451	0.0058	0.6205	-0.4828
PSMB8	0.1402	-0.0485	0.5759	-0.6198
PALLD	-0.3766	0.6196	-0.2969	-0.0368
CCDC109B	0.0747	0.1018	0.4531	-0.6192
C1S	-0.2295	0.5036	0.324	-0.6184
SOD2	0.0177	0.1279	0.5008	-0.6182
F13A1	-0.2478	0.6171	-3.00E-04	-0.4595
LAMB1	-0.3122	0.6165	-0.4881	0.0468
CLEC4A	-0.2482	0.2483	0.6163	-0.52
MOXD1	-0.3953	0.6158	-0.0913	-0.1845
LY96	-0.3406	0.4849	0.5175	-0.6146
ITGA5	-0.3109	0.6143	-0.0121	-0.3625
S100A6	0.2178	0.1439	0.1641	-0.6129
TREM2	-0.0416	0.1758	0.5122	-0.6109

C1ORF114	-0.0345	-0.2167	-0.335	0.6108
GUCY1A3	-0.3036	0.6096	0.0504	-0.4199
COL9A3	-0.165	-0.0762	-0.3405	0.6088
OSMR	0.0517	0.2631	0.2375	-0.6087
OLR1	-0.1367	0.3708	0.3666	-0.6085
FCGR1A	-0.1796	0.2692	0.5823	-0.608
HCP5	-0.0442	-0.0236	0.6077	-0.4354
MMP9	-0.3067	0.2399	0.6052	-0.4224
TLR1	-0.0422	0.1953	0.4755	-0.6032
RGS4	-0.2501	0.6025	-0.1469	-0.3114
ACSL5	0.3551	-0.0965	0.2969	-0.6022
VNN2	0.0344	-0.0386	0.5995	-0.513
HLA-F	-0.0066	0.0618	0.5982	-0.5859
LHFP	-0.2531	0.5979	-0.2281	-0.2316
FSTL1	-0.3939	0.5962	-0.3895	0.0958
CDR1	-0.2822	0.5961	-0.4107	-0.0335
MFAP2	-0.5955	0.4204	-0.3496	0.5522
LGALS9	0.12	0.0283	0.4631	-0.5939
TMEPAI	-0.1779	0.5931	-0.4226	-0.1573
SERPINB1	0.1432	0.0648	0.3707	-0.5916
TMEM140	0.0348	0.074	0.5232	-0.5914
TGFB1I1	-0.2319	0.5905	-0.2287	-0.2498
TLR3	0.1925	0.0105	0.374	-0.5902
SALL2	-0.2703	-0.0266	-0.2304	0.5898
TLR7	-0.1362	0.2662	0.499	-0.5897
MFAP5	-0.2063	0.587	-0.3261	-0.195
NID2	-0.2288	0.5856	-0.3115	-0.1761
CALD1	-0.1796	0.5841	-0.3723	-0.1869
CYR61	-0.1552	0.5829	-0.4534	-0.1478
IFI35	-0.0502	0.0359	0.5822	-0.4813
S100A9	0.1013	0.1566	0.2879	-0.582
HCLS1	-0.1466	0.243	0.5393	-0.5811
CDCP1	0.1783	0.0842	0.2749	-0.58
RGS16	-0.2756	0.5786	-0.2164	-0.1873
F2R	-0.4247	0.5745	-0.0859	-0.0975
MUC16	0.3219	0.0933	0.0274	-0.5683
CILP	-0.2167	0.5662	-0.3145	-0.1648
KLK7	0.2076	0.073	0.2287	-0.5647
HSD17B6	-0.3751	0.564	-0.1018	-0.136
IL18	0.0702	0.1691	0.2956	-0.5633
MEST	-0.2375	-0.0207	-0.2575	0.5624
PDGFRA	-0.1845	0.5617	-0.2701	-0.2399
FBN2	-0.3424	0.1143	-0.2942	0.5603
RARRES1	0.0545	0.2274	0.2277	-0.5584
CD248	-0.3347	0.5581	-0.1797	-0.1147
CLEC5A	-0.0628	0.2564	0.3633	-0.5573

CP	0.1779	0.0379	0.3146	-0.5547
HMOX1	-0.1479	0.2839	0.4468	-0.5519
OLFML3	-0.373	0.5501	-0.0138	-0.1969
PYCARD	0.0069	0.1726	0.3718	-0.5499
CASP4	0.1318	0.1676	0.1861	-0.5485
CD36	-0.3637	0.5479	0.0419	-0.2545
VAV1	0.0315	0.1503	0.3647	-0.5477
LGALS1	-0.2364	0.5464	0.1376	-0.5033
DRAM	-0.1306	0.3268	0.3484	-0.5447
SLC31A2	-0.178	0.2912	0.4725	-0.5436
SLC7A7	-0.2996	0.2856	0.5432	-0.4367
KRT23	0.1935	0.1985	0.0394	-0.5431
XAF1	0.0868	0.0553	0.4125	-0.5409
CTGF	-0.1649	0.5398	-0.3458	-0.1726
GJA1	-0.2882	0.5383	-0.0099	-0.2973
COL4A1	-0.3569	0.538	-0.161	-0.0759
ADORA3	-0.0772	0.1575	0.5058	-0.5349
ANXA1	0.1985	0.2432	-0.0446	-0.5342
GLRX	-0.2383	0.3006	0.5324	-0.5274
ICAM1	-0.0841	0.3007	0.3009	-0.5319
SLC34A2	0.1979	0.1313	0.1189	-0.5316
LHX1	-0.1589	-0.0448	-0.3062	0.5311
DAPP1	0.1249	-0.0016	0.427	-0.5311
EHF	0.3644	-0.0943	0.1957	-0.53
MUC1	0.2338	0.0363	0.2025	-0.53
HTRA1	-0.0814	0.5291	-0.3144	-0.2965
HEG1	-0.1986	0.5284	-0.1724	-0.2631
SAT1	0.0941	0.1548	0.2368	-0.526
DAB2	-0.2358	0.5259	0.1625	-0.4994
HMHA1	0.0174	0.1349	0.3817	-0.5242
SLC46A3	0.0405	0.2075	0.2373	-0.5229
IGLV2-14	-0.3407	0.2193	0.5216	-0.2791
CFH	-0.0513	0.4483	0.0176	-0.5194
LAMA4	-0.2061	0.5183	-0.1066	-0.2969
TNFSF4	-0.3854	0.5177	-0.0151	-0.1382
IFIT2	0.1643	0.0112	0.3317	-0.5174
PTGER2	0.1736	0.2386	-0.02	-0.5166
FAM129A	0.0545	0.1193	0.3388	-0.5163
MXRA8	-0.3721	0.5161	-0.3294	0.1175
PTRF	-0.1728	0.5159	-0.333	-0.1427
IGFBP4	-0.3069	0.5152	-0.2494	-0.0365
HPR	0.1984	0.027	0.2521	-0.514
ISLR	-0.2167	0.5138	-0.2019	-0.1951
IFIH1	0.1481	-0.0596	0.4569	-0.5136
SECTM1	0.1566	0.0567	0.2692	-0.5114
FER1L3	0.1685	0.2236	0.0035	-0.5109

OASL	0.0237	-0.0241	0.5094	-0.4397
CH25H	-0.1533	0.5079	-0.0309	-0.4188
C5ORF13	-0.4488	0.2549	-0.2772	0.507
MXRA5	-0.1937	0.5061	-0.2962	-0.1344
TNFAIP8	0.0475	0.0077	0.4996	-0.5033
HTR3A	0.2335	-0.0464	0.2927	-0.5017
RFTN1	0.1176	0.1632	0.1599	-0.5015
PLAUR	-0.1928	0.4089	0.2706	-0.5001
NUPR1	0.0798	0.1172	0.2821	-0.4983
UBE2L6	0.0444	-0.008	0.4982	-0.478
KIF1A	-0.2494	-0.0295	-0.1504	0.4969
VNN1	0.3079	-0.0417	0.1653	-0.4964
ITM2C	-0.2208	0.0162	-0.2606	0.4959
HLA-DOB	0.0667	-0.0986	0.4947	-0.3889
SLC28A3	0.1866	0.0333	0.2353	-0.4919
IL1B	-0.0623	0.1855	0.3905	-0.4909
HERC5	-0.0469	-0.1067	0.4909	-0.2249
PAPSS2	-0.2004	0.4893	-0.0381	-0.3265
HP	0.1706	0.0689	0.2029	-0.4884
S100A8	0.053	0.1703	0.2327	-0.488
APOE	-0.2768	0.1792	0.4879	-0.2833
KLHL23	-0.1093	-0.2543	-0.0208	0.4868
TRPS1	0.1311	0.1249	0.1784	-0.4865
CTA-246H3.'	-0.3185	0.2134	0.4861	-0.2703
CST7	-0.2602	0.3961	0.3758	-0.4854
LTB	-0.1547	0.1311	0.4852	-0.3811
FXYD1	-0.0802	-0.2016	-0.1408	0.4846
SLC12A8	-0.1676	0.4845	-0.1049	-0.3062
CNN1	-0.2669	0.4842	-0.2517	-0.048
TGFB3	-0.3079	0.4841	-0.3593	0.099
SNX10	-0.3083	0.257	0.484	-0.3376
THBS4	-0.107	-0.0842	-0.2728	0.484
GPX3	0.0556	0.1507	0.2515	-0.4827
PDGFD	-0.2117	0.4819	-0.2523	-0.1175
RGS2	-0.2919	0.4819	0.0108	-0.2383
P2RY5	-0.1053	0.2714	0.3176	-0.4808
CNN2	-0.1772	0.4795	-0.0889	-0.3009
STK17B	-0.0735	0.2522	0.2952	-0.479
BCL2A1	-0.2258	0.2666	0.4787	-0.4543
OAS1	0.1212	-0.0247	0.4051	-0.478
ARNT2	-0.1785	-0.028	-0.2387	0.4775
LY75	0.2625	-0.1608	0.3887	-0.4768
SEMA3C	-0.0682	0.4768	-0.2437	-0.3082
ENPP1	-0.2658	0.4752	-0.2568	-0.0335
IGF2BP3	-0.2189	-0.1276	-0.0261	0.4746
EPB41L3	-0.2591	0.3615	0.4128	-0.4746

GPC3	-0.2403	0.0111	-0.1981	0.4743
TNFAIP2	0.2121	-0.0068	0.2344	-0.4738
TMEM45A	-0.4555	0.473	-0.3175	0.2724
TRO	-0.2519	0.0346	-0.213	0.4724
CYP4B1	0.3451	-0.1722	0.2738	-0.4724
PLAC8	0.0099	-0.0795	0.4716	-0.3183
IFI27	0.1171	-0.0109	0.3829	-0.4709
GPR64	0.0088	0.1892	-0.4702	0.1523
THY1	-0.2151	0.4697	-0.2798	-0.0737
DDR2	-0.2642	0.4691	-0.1714	-0.1016
INPP4B	0.0169	0.2228	0.1879	-0.4687
TNC	-0.2192	0.4678	-0.0494	-0.2645
VAV3	0.1478	0.0231	0.2814	-0.4674
CLIC2	-0.1875	0.2411	0.4662	-0.4616
DUSP10	0.0636	0.167	0.1956	-0.4657
PLS3	-0.1394	0.4651	-0.1542	-0.2762
TNFAIP3	-0.0567	0.2573	0.2452	-0.4647
APOBEC3A	-0.1239	0.0307	0.4645	-0.2757
STAT1	-0.1122	-0.0058	0.4644	-0.2447
CX3CR1	0.1303	0.1184	0.1636	-0.4643
ACSL1	-0.1221	0.4331	0.0841	-0.4637
CHI3L1	0.2311	-0.094	0.3223	-0.4635
TTYH1	-0.0865	-0.1722	-0.1496	0.463
ELTD1	-0.279	0.4628	0.0304	-0.2479
A2M	-0.063	0.2148	0.3153	-0.4626
HPSE	0.0183	0.0278	0.4613	-0.4574
PLSCR1	0.0254	0.0136	0.4613	-0.4486
ATP8B1	0.2396	-0.0114	0.184	-0.4608
RSAD2	-0.0188	0.1168	0.3897	-0.4601
LXN	-0.0374	0.4012	-0.0041	-0.4589
CYP1B1	-0.1675	0.4585	-0.035	-0.3334
ANPEP	0.1797	0.0665	0.1571	-0.4577
SLIT3	-0.2203	0.2424	-0.457	0.3758
LRRC17	-0.2861	0.4561	-0.3991	0.1403
PKIA	-0.2681	0.0852	-0.2436	0.4558
LAP3	-0.0691	0.0384	0.4554	-0.3502
RHOBTB1	-0.2856	0.1639	-0.3325	0.4551
BHLHB2	0.028	0.3556	-0.0418	-0.4546
MARCKS	-0.4537	0.3873	0.1304	-0.0068
TFPI2	0.0664	0.2313	0.0805	-0.4524
CD44	-0.038	0.1668	0.3359	-0.4522
RARRES2	-0.1941	0.4514	-0.214	-0.135
IL32	-0.0964	0.2552	0.2934	-0.4511
NTF3	-0.2265	0.0434	-0.2397	0.4507
TFAP2C	0.2454	0.059	0.0577	-0.4493
AHR	0.1493	0.1376	0.0879	-0.4486

MFAP4	-0.3301	0.4479	-0.4056	0.2147
ADAMTS5	-0.2374	0.4469	-0.3524	0.0473
RAB27A	-0.0209	0.104	0.3965	-0.4468
GCH1	-0.2006	0.0335	0.4468	-0.1627
CBR3	0.1823	-0.0494	0.3116	-0.4466
DPYSL3	-0.1384	0.4462	-0.1859	-0.2261
KCTD12	0.1223	0.1754	0.07	-0.4459
MAFB	-0.2483	0.4456	0.1863	-0.401
FLRT3	-0.1424	0.0143	-0.3194	0.4454
LCP1	-0.1741	0.1904	0.4444	-0.396
MLLT11	-0.3843	0.2403	-0.2813	0.4439
EFEMP1	-0.2062	0.4435	-0.0366	-0.2617
FGF9	-0.0848	0.0074	-0.3953	0.4433
JAM2	-0.176	0.4427	-0.2672	-0.1018
BCL3	0.1102	0.0996	0.1966	-0.4423
SLC15A3	-0.0044	0.0586	0.4325	-0.4419
SPON1	0.0756	0.1592	0.1608	-0.4417
PLK2	-0.0715	0.4402	-0.3872	-0.1334
PDGFRL	-0.1419	0.4398	-0.1682	-0.2285
PGDS	0.006	0.1544	0.2707	-0.4383
C1ORF165	-0.2294	0.0104	-0.1718	0.438
DSE	-0.3131	0.4374	0.1314	-0.2575
SLIT2	-0.2336	0.3785	-0.437	0.2026
PCSK5	-0.239	0.4368	-0.3068	0.0231
MMP1	-0.2943	0.4344	-0.0873	-0.09
FBXO17	-0.1388	-0.0873	-0.1608	0.4335
C10ORF116	0.3534	0.0062	-0.0487	-0.4331
LAMP3	0.0357	-0.1042	0.4327	-0.2873
STEAP1	0.0725	0.1733	0.1335	-0.432
CCND1	0.0153	0.1685	-0.4296	0.1352
FAS	-0.0732	0.3029	0.1616	-0.4289
OAS2	0.027	0.0274	0.414	-0.4276
NDRG1	0.1261	0.1309	0.1068	-0.4257
FBLN1	-0.2292	0.4248	-0.2653	-0.0102
KIAA0888	-0.1535	-0.077	-0.1433	0.4246
PCP4	-0.0087	-0.0096	-0.4243	0.3896
CHODL	0.0696	-0.1066	0.4239	-0.3215
CXCL1	0.0784	0.1978	0.0787	-0.4239
BTN3A2	0.0842	-0.0614	0.423	-0.3976
TMEM2	-0.0907	0.4227	-0.129	-0.3082
SPOCK1	-0.3212	0.4224	-0.355	0.1917
GABRE	0.0313	0.095	0.3012	-0.4223
CSRP2	-0.3341	0.1364	-0.1787	0.4216
MT1E	0.0468	0.1337	0.2192	-0.4214
IFI44L	0.058	-0.0247	0.4212	-0.4083
LGALS3	0.2498	0.0089	0.0923	-0.4212

CTSO	-0.0748	0.2356	0.2541	-0.4207
COL15A1	-0.2262	0.4202	-0.1666	-0.0935
TM4SF1	0.1507	0.0901	0.1221	-0.4195
BMP4	-0.1859	0.4192	-0.3689	0.0289
CHN1	-0.3398	0.4192	-0.0307	-0.0592
FAM105A	-0.1353	0.0881	0.4185	-0.2943
FLJ20035	0.0328	0.001	0.4184	-0.4053
IFI44	0.0415	-0.0518	0.417	-0.3483
ZFP36	0.0883	0.256	-0.0323	-0.4155
WASF1	-0.1623	0.0125	-0.2514	0.4153
CDH2	-0.1651	0.1053	-0.3844	0.4153
OVGP1	0.4153	-0.3092	0.0579	-0.2044
PXDN	-0.14	0.4149	-0.3731	-0.0226
GRAMD3	0.1284	0.1422	0.0733	-0.4143
KLK10	0.1457	0.0903	0.123	-0.4139
TMPRSS4	0.1349	0.0878	0.1425	-0.4131
IFITM1	0.1081	0.0457	0.2449	-0.4123
CCL20	0.1532	-0.0113	0.2591	-0.4116
EGR2	-0.1582	0.411	-0.0767	-0.2492
KLK8	0.159	0.0311	0.1862	-0.4104
PRKD1	-0.2847	0.4102	-0.1055	-0.0561
MARCKSL1	-0.2624	0.0135	-0.0932	0.41
ACP5	-0.1264	0.0968	0.4098	-0.3098
CLUL1	-0.1164	-0.1661	-0.0508	0.4096
BAMBI	-0.1027	0.0486	-0.3888	0.4089
KAZALD1	0.0064	-0.1991	-0.1895	0.4089
COCH	-0.1079	-0.2183	0.0145	0.4087
RP4-691N24	-0.1108	-0.0707	-0.1991	0.4083
VDR	-0.0289	0.328	0.0323	-0.4081
BTN3A3	0.0783	-0.0444	0.4071	-0.3979
AIM1	0.0758	0.0716	0.2498	-0.4068
COL4A2	-0.2785	0.4063	-0.2082	0.0291
FBP1	0.0489	0.0326	0.3477	-0.406
KDELR3	-0.032	0.4059	-0.1678	-0.331
IGF1	-0.2279	0.4055	-0.1714	-0.0684
IGFBP5	-0.15	0.1939	-0.4055	0.3005
CD55	0.1133	0.139	0.0909	-0.4054
DHCR24	0.2313	-0.0348	0.1654	-0.4038
LPL	-0.2092	0.4036	-0.0615	-0.1854
SLPI	0.1907	0.0263	0.1367	-0.4035
BBOX1	0.2025	-0.0277	0.1982	-0.4031
MYH10	-0.2349	0.2409	-0.4026	0.3502
GADD45B	0.0089	0.4022	-0.1497	-0.396
ALPL	-0.1137	-0.1066	-0.1344	0.402
MX1	0.1277	-0.0409	0.331	-0.4019
PNMA3	-0.0077	-0.2028	-0.1527	0.4006

AHNAK2	0.1327	0.2415	-0.0967	-0.4001
LTBP2	-0.1222	0.3994	-0.1946	-0.1802
FMO2	-0.0935	0.3062	0.1525	-0.3986
SMPDL3A	-0.2827	0.354	0.3717	-0.3984
SPARCL1	-0.2132	0.3978	-0.0683	-0.1668
ITGAV	-0.0406	0.2295	0.1839	-0.3976
IGF2	-0.2141	0.2739	-0.3965	0.2754
PLVAP	-0.2121	0.3965	-0.0687	-0.1663
MGLL	0.2173	0.1024	-0.028	-0.3938
MMP12	-0.3108	0.2369	0.3936	-0.2307
BIK	0.1805	-0.0162	0.2033	-0.3932
VIM	-0.3184	0.3918	-0.1018	0.0088
TSPAN8	0.2151	-0.3917	-0.1028	0.3041
TSPAN7	-0.238	0.1873	-0.3664	0.3914
ST3GAL1	0.0735	0.2472	-0.0258	-0.3903
DHRS3	0.2712	-0.2017	0.3351	-0.3901
SDC1	-0.2977	0.3898	-0.2236	0.089
TIMELESS	-0.2071	-0.1467	0.0837	0.3889
C9ORF95	0.1224	0.0888	0.1317	-0.3886
MMP7	0.146	0.2107	-0.0857	-0.388
RNASE1	-0.0593	0.0912	0.388	-0.3726
BST2	0.1571	-0.0213	0.2404	-0.3878
RHOBTB3	-0.3829	0.3875	-0.3377	0.3028
SGMS1	0.2005	-0.039	0.1995	-0.3872
B3GNT3	0.2033	0.0601	0.0484	-0.3872
C20ORF103	-0.1467	0.2755	-0.3871	0.1761
SAMHD1	-0.058	0.0163	0.3861	-0.277
BST1	0.0091	0.1524	0.2078	-0.3855
INA	-0.194	-0.0081	-0.1373	0.3852
SAMD9	0.1277	-0.0066	0.2606	-0.3851
HTATIP2	0.1392	-0.0779	0.3465	-0.3833
TMEM176B	-0.2906	0.3821	0.3206	-0.3798
MYO1B	-0.3821	0.3416	-0.2036	0.2447
NOX4	-0.2457	0.3819	0.1312	-0.2756
SDC2	-0.1711	0.3456	-0.38	0.1128
CRYAB	0.1939	0.1858	-0.1337	-0.3781
GAS1	-0.1405	0.3776	-0.1085	-0.2025
PLCB4	-0.1467	0.0367	-0.2665	0.3768
RAB38	-0.0353	-0.1439	-0.1692	0.376
RAI14	-0.2721	0.3759	-0.2303	0.0786
NUDT11	-0.2592	0.1682	-0.2872	0.3756
NPR1	0.2772	-0.0534	0.0896	-0.3755
UCHL1	-0.3755	0.0617	0.0627	0.3635
EMX2	0.0476	0.0273	-0.3754	0.226
IRF7	0.1204	-0.0175	0.2761	-0.3748
LOH11CR2A	0.1967	0.0143	0.1117	-0.3745

BACE2	0.2357	-0.0221	0.1057	-0.3745
CXCL2	0.0968	0.188	0.0075	-0.3743
DOK5	0.1693	0.0153	0.1513	-0.3737
IL11RA	-0.1738	-0.0294	-0.1228	0.3731
CSTA	0.0174	0.0357	0.3525	-0.3725
DUSP1	-0.0012	0.3715	-0.1985	-0.3012
OAS3	0.0752	-0.0538	0.3714	-0.3509
HRH1	0.1907	0.1686	-0.1116	-0.371
CLU	0.3701	-0.1599	-0.0697	-0.2251
TAPBPL	0.1295	-0.0951	0.3698	-0.3686
FRAS1	-0.1362	-0.0224	-0.1868	0.3697
ARHGAP26	0.2995	-0.179	0.2341	-0.3693
DDIT4	0.0225	0.1608	0.1554	-0.3688
LOC93349	0.1319	-0.0316	0.2722	-0.3687
DNAJC15	0.0307	0.1337	0.1827	-0.3686
GHR	-0.2145	0.3685	-0.1657	-0.0438
PSTPIP2	-0.1047	0.1789	0.3216	-0.3671
TREM1	-0.1827	0.3014	0.2598	-0.3671
GALNAC4S-	-0.1114	0.3669	-0.1614	-0.1818
CLDN10	0.1915	-0.0294	0.1752	-0.3667
EMP1	-0.0783	0.366	-0.2779	-0.124
GLI3	-0.2025	0.3467	-0.3653	0.1402
GPR177	-0.1281	0.0369	-0.2812	0.3647
MLPH	0.3639	-0.1462	-0.0204	-0.2768
SLC2A3	-0.239	0.3635	0.0122	-0.1583
ST6GALNAC	0.1232	0.2278	-0.1047	-0.3632
IER3	0.0212	0.2294	0.0489	-0.3628
JUNB	0.1247	0.1934	-0.0569	-0.3627
KLF6	0.0152	0.2841	-0.0243	-0.3616
SKAP2	-0.0489	0.2347	0.146	-0.3606
PDE1A	0.0811	0.1896	0.0126	-0.36
GNG11	-0.1536	0.3596	-0.1823	-0.0986
KDELC1	-0.359	0.3554	-0.1827	0.1786
C13ORF15	-0.0543	0.3269	0.016	-0.359
CDH3	-0.1249	-0.09	-0.0914	0.3588
KLK6	0.0733	0.2293	-0.0358	-0.3586
SGK	-0.0587	0.2706	0.1051	-0.3583
SLC5A1	0.1965	-0.1019	0.2634	-0.3567
AQP9	0.175	0.0643	0.0496	-0.356
CDC7	-0.1891	-0.1239	0.0604	0.356
FOS	0.1243	0.3174	-0.2526	-0.3515
IFIT1	0.0194	-0.038	0.35	-0.2789
PAM	-0.201	0.179	-0.3499	0.3389
SCG5	-0.0406	-0.0604	-0.2535	0.3493
FMOD	-0.0782	0.1256	-0.349	0.244
KLK11	0.1535	0.0582	0.0817	-0.3475

CA12	0.123	0.0448	0.1461	-0.3456
FLRT2	-0.1592	0.3378	-0.3453	0.0771
TMEM176A	-0.2524	0.345	0.2348	-0.309
CDK6	-0.1336	0.1479	-0.3448	0.2851
S100A14	0.2847	-0.0594	0.0505	-0.344
SOAT1	-0.1887	0.1481	0.3437	-0.2359
LIPA	-0.1454	0.1196	0.343	-0.256
FBXO2	-0.0916	-0.0422	-0.1939	0.342
CLGN	-0.0491	-0.0583	0.3418	-0.1554
LY6E	0.16	-0.0276	0.1912	-0.3412
HRASLS3	0.289	-0.0881	0.0822	-0.3405
AMIGO2	-0.0726	0.3399	-0.0964	-0.2546
PRRG4	0.2938	-0.193	0.2291	-0.3398
EGFL6	-0.3079	0.3397	-0.1752	0.1246
BLNK	-0.0071	0.0176	0.3392	-0.3055
TCAG7.1314	0.1996	-0.0791	0.2043	-0.3391
EDG2	-0.1847	0.339	-0.0754	-0.1234
CKB	0.0031	-0.1592	-0.1618	0.3384
MYLK	-0.1008	0.3372	-0.0698	-0.2369
CYP2J2	0.1329	-0.1956	0.3361	-0.2158
SERPINA3	0.1156	0.0222	0.1787	-0.3351
NQO1	0.2021	-0.1447	0.2928	-0.335
ADFP	-0.3341	0.1406	0.228	0.0657
WNT7A	-0.0968	0.3327	-0.25	-0.081
IGF2BP2	-0.1007	-0.0435	-0.167	0.3325
FYN	-0.1771	0.058	-0.1997	0.3322
NDN	-0.1858	0.3321	-0.268	0.0528
PI3	0.1075	0.0562	0.1368	-0.3317
MT1G	0.0347	0.0276	0.2905	-0.3315
POU2F3	0.2352	-0.1303	0.2159	-0.3308
NEFH	-0.109	-0.0791	-0.0991	0.3304
TCEAL2	-0.1779	0.0497	-0.1838	0.3302
FAT	-0.0747	0.3301	-0.179	-0.168
NAP1L3	-0.2027	0.2114	-0.3299	0.2826
TNFRSF11B	0.1819	0.0632	0.0099	-0.3297
MGAT4A	-0.1924	0.2185	0.3297	-0.3087
HSPA1A	0.046	0.1249	0.1271	-0.3297
GINS1	-0.2067	-0.1223	0.1158	0.3294
SOSTDC1	0.0187	0.0621	-0.3293	0.1799
BTC	0.0963	-0.0765	0.3285	-0.3129
EGR1	0.1479	0.2678	-0.2423	-0.3282
FADS1	-0.3278	0.17	-0.0643	0.2718
VGLL1	0.2312	0.109	-0.136	-0.3276
CPZ	-0.1184	0.3262	-0.2633	-0.0326
ATP10B	0.2423	-0.0924	0.1435	-0.3261
KAL1	-0.163	0.3252	-0.0054	-0.1948

PRSS23	-0.0698	0.3248	-0.2209	-0.1317
EGLN3	0.0183	0.1886	0.0693	-0.3246
TSPAN12	0.1223	0.1337	-0.0092	-0.3243
ID1	-0.1962	0.2483	-0.3226	0.2207
RAB32	-0.2217	0.1759	0.3223	-0.2092
RAD51AP1	-0.2456	-0.1146	0.1752	0.3198
ADM	-0.0862	0.2823	0.0803	-0.3154
NEDD9	-0.1336	0.1426	-0.3147	0.266
CEACAM6	0.121	-0.1374	0.3145	-0.2558
PEG10	-0.0026	-0.1886	-0.0814	0.3142
C1ORF116	0.2971	0.0221	-0.1243	-0.3139
GPX7	-0.2525	0.1115	-0.1419	0.3138
EFNB2	-0.1201	0.1753	-0.3121	0.2042
CPE	-0.2032	0.3121	-0.2043	0.0465
KIAA1199	-0.1043	0.3115	-0.2976	-0.0031
S100A10	-0.0703	0.3114	-0.1531	-0.1724
GRAMD1C	0.3114	-0.2646	0.0377	-0.1065
NETO2	-0.3112	0.1089	-0.0232	0.2924
ACPP	0.1145	-0.1067	0.3104	-0.2828
KCNJ8	-0.3104	0.2998	-0.0335	0.0566
UBB	-0.0439	0.1634	0.1852	-0.3101
GJB1	0.2268	-0.1407	0.219	-0.3092
LRAP	-0.022	0.1078	0.2324	-0.3089
PLEKHF1	0.1084	-0.0714	0.2978	-0.3089
EFEMP2	-0.2283	0.3087	-0.2751	0.1451
TNNC1	-0.0716	0.166	-0.3084	0.1488
ISG15	0.0563	-0.0213	0.3027	-0.3082
COBL	0.308	-0.2952	0.0276	-0.0544
ZBTB16	0.1828	-0.035	0.1256	-0.3052
PLAT	-0.1349	0.3047	-0.1788	-0.0564
FSCN1	-0.3045	0.2759	-0.2718	0.2848
DOCK4	-0.1969	0.3043	0.0849	-0.2012
NTS	-0.0868	-0.1252	0.3041	0.0124
STXBP6	0.0517	-0.0734	0.3004	-0.2337
QPCT	-0.0514	0.1585	0.1925	-0.3002
DDX58	0.0872	-0.0461	0.2826	-0.3001
SEMA5A	-0.1315	0.2993	-0.2687	0.0236
HERC6	0.1039	-0.0532	0.2651	-0.2981
SPRY2	-0.0811	0.088	-0.2977	0.2515
GSTM3	-0.0635	0.018	-0.2745	0.2976
TMEM100	-0.1473	0.2038	-0.2975	0.191
CRTAC1	0.0496	-0.0931	-0.2821	0.2963
FRZB	-0.1865	0.0427	-0.1187	0.2944
FOSB	0.0572	0.2703	-0.2944	-0.1667
SCARA3	0.2941	-0.1007	-0.0389	-0.2267
IL8	0.0034	0.136	0.1331	-0.2927

MYO5C	0.2909	-0.1669	0.0514	-0.216
PPIC	-0.0647	0.2908	-0.0146	-0.2729
CDKN2A	0.1032	-0.1261	0.2905	-0.226
HOXA5	-0.0984	0.2904	-0.1668	-0.0966
FXYD3	0.2825	-0.1734	0.1584	-0.2887
TMEM47	-0.0914	0.2343	-0.2886	0.0706
SEMA3A	-0.0958	0.1764	-0.2882	0.15
CLDN16	-0.0387	0.2714	-0.0092	-0.2872
ITGB4	0.2545	0.0633	-0.1517	-0.2863
TOP2A	-0.2801	-0.0576	0.1829	0.2861
D4S234E	0.0354	-0.1346	-0.1867	0.2858
PRUNE2	0.2849	-0.1016	-0.0778	-0.1799
S100A1	0.2284	-0.0545	0.0573	-0.2817
FBLN5	-0.1485	0.2814	-0.2103	0.0185
PLOD2	-0.1648	0.2813	0.0331	-0.1697
DZIP1	-0.1842	0.1399	-0.2492	0.2799
MALL	-0.0234	0.2644	-0.0311	-0.2795
ZBED2	0.1641	0.1189	-0.1035	-0.2794
TMC5	0.2601	-0.1918	0.208	-0.2784
TPPP3	0.0827	-0.1108	-0.2772	0.2711
ZFPM2	-0.0556	0.277	-0.1614	-0.1408
MMD	-0.2279	0.2766	0.1846	-0.2108
NLGN4X	-0.1577	0.1907	-0.2766	0.2036
FOLR1	0.2762	-0.1729	0.0374	-0.1767
MYB	0.1458	-0.274	0.2669	-0.0731
RAB25	0.274	-0.0365	-0.0641	-0.2604
MGP	-0.0728	0.2738	-0.0876	-0.1776
PRSS16	0.2266	-0.1315	0.1645	-0.2735
ATP6V1B1	0.2735	-0.0949	-0.0792	-0.1722
TEKT2	0.1475	-0.2719	-0.1115	0.2481
TMPRSS3	0.2716	-0.1937	0.1011	-0.1991
PROS1	0.0146	0.2714	-0.1933	-0.1989
TYMS	-0.2659	-0.0744	0.2706	0.2132
GUCY1B3	-0.1658	0.161	0.2704	-0.2192
CASC1	0.0678	-0.1888	-0.1368	0.2691
MAP1B	-0.1802	0.1611	-0.2667	0.2625
ID4	0.118	-0.1881	-0.2106	0.2656
UPK1B	-0.0667	0.2655	-0.1643	-0.1088
S100A2	0.0311	0.1964	-0.0308	-0.2652
IGFBP2	-0.1478	0.0866	-0.2089	0.265
ATP10D	-0.1822	0.265	0.1385	-0.2166
ITM2A	-0.2643	0.204	0.2115	-0.0933
PTH2R	-0.1555	9.00E-04	-0.0675	0.2627
PCOLCE2	-0.2626	0.232	-0.1928	0.2173
DEPDC6	0.2618	-0.1893	0.0789	-0.1726
SPDEF	0.261	-0.2423	0.11	-0.1306

MSLN	0.2592	-0.047	-0.0307	-0.2562
FZD7	-0.1953	0.2131	-0.2587	0.2092
WNT11	-0.1815	0.1793	-0.2552	0.2311
C11ORF75	-0.1703	0.255	0.1568	-0.2354
TUBA1A	-0.2364	0.1821	-0.2025	0.2547
LTF	0.07	-0.0065	0.1962	-0.2534
SCGB2A1	0.1969	-0.0615	0.0826	-0.253
GPRC5A	0.0944	0.1961	-0.1475	-0.2479
KLHDC8A	0.1546	-0.246	-0.1349	0.2258
PTGDS	-0.1467	0.2458	0.0603	-0.1717
WISP3	0.1623	-0.2018	0.2455	-0.1687
SLC44A4	0.1904	-0.1396	0.1993	-0.2454
SELENBP1	0.2154	-0.2395	0.1976	-0.1495
CD302	-0.205	0.239	0.1977	-0.2045
LMO3	-0.1276	0.0568	-0.157	0.2314
FOXA2	0.141	-0.2058	-0.1786	0.2302
SNCG	0.2238	-0.1408	0.1244	-0.2234
ZIC1	-0.199	5.00E-04	0.0605	0.2102
OXTR	0.1766	0.1019	-0.1916	-0.1982

Supplementary Table S2
Signature genes of the Mesenchymal and Immunoreactive subtypes

Mesenchymal	Immunoreactive
OXT	DOK3
GPR4	PIK3R5
MAB21L2	FASLG
SEPT4	TRGV7
TRPC3	NAT8B
FAM65A	SIGLEC7
LL22NC03-75B3.6	WAS
MS4A2	SLC11A1
DAPK3	MAP3K14
ADAMTSL2	FAIM3
SHC1	FKBP15
BTN1A1	TFIP11
FKBP15	IL16
PIK3CD	GPR132
FBXW7	CLN3
SYDE1	FLT3LG
LAT2	EDEM1
RAPGEF4	CCDC134
VASH1	DENND3
SV2B	CSF2RB
TAX1BP3	CD40LG
TSPAN5	KIAA0748
AZI2	LILRA1
ARHGDIA	RHBDF2
MAP1A	MTCH2
TSPAN9	CCDC69
SNTB2	STK10
RAP2B	C22ORF9
RASAL2	TRA@
GRB2	TRADD
FTO	SOCS1
NDEL1	DNPEP
CARD8	MICAL1
ARL2BP	PTPN2
PPP3CC	GPBP1L1
RAP2A	MED8
ZNF281	MYO7A
CCPG1	DSCR3
KIAA0427	COMMD9
GPR68	IL12RB1

VKORC1	SP100
BACH1	CAPZA1
IL1RL1	GZMM
RIN3	MFNG
TIE1	MRPL16
GNA15	GRB2
PRRC1	RNF185
ASPH	MBD4
HERC3	CREM
EDEM1	ENTPD1
NAGA	GTF2E1
ABHD2	CLIC1
COBLL1	RNF13
EHD3	ENOPH1
ABL2	SLC33A1
NUCB1	MYD88
TNFRSF10D	KCNMB1
PTGDR	ARPC3
GLS	IRAK1
ACVRL1	IL17RA
DOCK10	STX11
ACAN	XRCC6
HABP4	TRIM26
SEC24A	UTP6
GNAL	AMPD3
TSPAN4	OTUD4
RPS27L	ATOX1
CLTC	MLX
ARMC9	ZC3HAV1
SH3GL1	MEFV
LILRB2	TGFB1
GRIA3	PPP4C
CAST	NSUN3
PHLDB1	PLA2G2D
BMPR2	KIR2DL4
EHD4	STX12
KIAA0513	CASP5
STAT3	LNPEP
TCF12	ZEB2
M-RIP	POLR1C
ATP6V1D	FTL
SMPD1	HMGN4
MTSS1	CD46
SIRPA	UEVLD
BEST1	PSMA1
C21ORF7	RAB5C

P2RY10	HCFC2
LILRB4	SLC25A17
ADARB1	HEXA
ITGAX	ATG7
ACTR2	UBE2A
PSAP	KCNJ10
MAPK6	SPN
REM1	MKNK1
HERPUD1	IFNAR2
IL21R	TMEM9B
CDC27	ATP5C1
IKZF1	SEC22A
EXTL1	CTSD
STK10	FTH1
P4HB	CYLD
TM9SF3	KIAA0082
SEC31A	C3ORF37
TBC1D2B	KLRC4
PTPN22	PTP4A2
HTR2A	BTG1
SLC36A1	NDUFAB1
RAB8B	MYO5A
SH3BGRL3	MTO1
FAM124B	MRPS10
MYO1C	RAP2B
RAB6IP1	UBE2D3
MYO5A	GM2A
ACTG1	AKR1A1
C11ORF17	PRDX1
SPAG9	PRDM1
ABR	TMED5
SNX24	NDUFB5
QKI	FAM50A
ERG	IFNGR2
ZCCHC6	APOBEC3F
DNM1	PCYT1A
SWAP70	BLVRA
PDGFB	PSMC2
BICD2	SSR1
ADAM10	SDHB
PLEKHQ1	DYNLT1
SLC22A3	GK
RNF19A	WTAP
KIAA0408	NDUFS3
SMTN	UCRC
SERINC1	USP15

KIAA1128	TRIM14
KIAA0802	DEF6
SNFT	CTSZ
TBXA2R	HK3
RAB23	INTS12
STK3	KATNA1
PLSCR3	NDUFS7
CDKL5	GAL3ST4
LSP1	POLR3K
UNC5C	PLCB2
FTL	HPS5
STX12	RHOG
PLA2G4C	PSMA4
RSU1	C5AR1
WWP1	CD47
TRPV2	C4ORF16
DENND2A	RENBP
CD300A	ATP6V1E1
CD4	MCM5
TMOD2	NINJ1
LNPEP	PISD
ARSB	RAP1A
MICAL1	CTSB
SERPINB8	CTBS
ARFGAP3	NFKBIA
MITF	SF3B5
CTSA	PTGER4
PIK3CG	ELF3
MEG3	SQSTM1
CYB5R3	GPX1
ATP8B2	NECAP2
MTMR6	ABI1
SEMA7A	CLDN7
BIN2	SCYE1
VAT1	GABARAPL2
UBL3	ADPGK
ABL1	SH3BGRL3
PICALM	LITAF
ACTR1A	MRPS30
DUSP3	RAD51
OSBPL8	LSM6
NBLA00301	SCO2
KITLG	TMEM111
C11ORF24	GTPBP2
MAPRE2	MCTS1
STARD8	ARRB2

C22ORF9	SGPP1
PLEC1	MED18
ITK	CCL23
GPR21	UBE2J1
GBE1	DMXL2
SLC12A4	IGHD
CASK	MPPE1
IMPAD1	GBAS
TMBIM1	SNFT
SAMD4A	OAZ1
CYP26B1	SYNGR2
CXORF36	ALG6
IL18R1	ABCD2
DYSF	CCND3
PCDH12	PCMT1
IL6ST	RBX1
ITGA2	CENPM
WWC3	ZNF267
EID1	PFN1
TOM1	METTL4
TPP1	GMDS
RNASEL	PARP3
MYO1E	TTRAP
RHBDF1	CDC45L
KLF9	GK3P
LILRB3	IKBKE
RAC2	DENND1C
ALOX5	HESX1
SELPLG	COQ2
TPM4	RELB
GPC1	CEPT1
VCL	NEIL3
RASSF4	MRPS18A
FAM49A	MRPL18
HIF1A	STAT3
RASL12	HEXB
CHRD	ADRBK2
CAPZB	NUP54
LST1	ARHGAP25
C10ORF26	EDG6
HIGD1B	NAIP
SGK269	TMEM62
WDR1	TRPM2
PANX1	GRAP2
FKBP10	PITPNB
PTGFR	COMMD4

ARL15	MR1
KCNMB1	LAT
RABGAP1L	MERTK
FOXN3	PSMB2
FNDC3B	ATP6V0D1
SLC6A6	HMGCL
TRAM2	GSTO1
SERPING1	RABGAP1L
CLEC11A	PSMA3
PDE4D	RHOA
FAM38A	FLVCR2
M6PRBP1	CENPI
RBM9	CD7
CLDN5	H2AFZ
AMPH	P2RY6
EVC	CORO1C
GRK5	FLOT1
RORA	TM6SF1
CXORF9	LYL1
MALT1	CBR1
PYCARD	IRF2
MAP3K5	CENTB1
SOX4	TMEM51
TGFBR1	ELL2
MGAT1	VAMP8
CD180	PGK1
CFHR2	STOM
APOE	ATG4A
CCR5	CUL2
ADCY7	MFN1
PLA2R1	TOR1B
CTSZ	HERPUD1
LIMS1	HLA-DRB6
HEXB	TSPO
RAB11FIP5	CCDC90A
CR1	CD83
ARNTL	PLEK2
FAM63B	BRP44L
GAL3ST4	TPP1
CASP4	OSTM1
TNFSF12	C17ORF42
PDLIM7	D15WSU75E
STAB1	AP1B1
ATP8B4	HMGB2
PTPRB	TMEM144
F2RL2	TK1

ACTB	LGMN
ECGF1	STAT5A
ZYX	PTPN6
PRRG1	PIP4K2A
WWC2	TRAF1
DDEF1	MEF2C
MYO1D	RAB8B
ARL6IP5	CSTB
SLC25A32	EIF2B3
TRPC1	TMEM50A
GOLM1	FMR1
LGMN	HHEX
TRPC6	ELMO1
C10ORF10	FAM49B
SOCS3	APOL2
GIMAP5	IL8RB
TBXAS1	PSMB3
LILRB1	PSAP
CD3E	GCLM
TM6SF1	FOLR2
SVIL	OGFRL1
CTSL1	SPCS3
IL13RA1	CCDC28A
MAPK9	LILRA2
RAB32	OSTF1
BACH2	CCL13
KLF7	PSMA6
MYO1F	PLEKHO1
PSCD4	PCAF
FGD6	C6ORF62
ITGAM	EBP
TUBB3	CALCOCO2
CLEC1A	MAT2B
SLC2A5	MAD2L1BP
TLR6	CD6
PELO	CHMP5
PRKACB	TXNL4B
DIP2C	AK2
CEP170	SLC25A5
EPHA3	BMP2K
PSTPIP2	COTL1
CCDC88A	ICAM2
ARSE	KIF18A
MID2	STIL
SH3GLB1	LY9
PTTG1IP	PCK2

TLE4	DENND2D
IRF8	MYNN
CD84	TNFRSF11A
LMNA	IDI1
MPP1	ACP2
LEPROT	SPI1
CYB5R4	COMMD3
GYPC	RTN1
CLEC1B	NIT2
BAG3	IMPA2
SGPP1	E2F8
PCDH7	IL1RN
RNASE2	ADRB2
ARID5B	SDF2L1
TUBA1A	IFIT5
COTL1	PHF11
TPST2	TRIM38
FOSL2	TAPBP
DKK3	BCL2L14
HGF	GMNN
CDC42EP3	TMEM165
C16ORF45	SERPINB8
SFXN3	SEMA4A
ARPC1B	SHCBP1
ZNF659	CRLF3
UPP1	MOBKL1B
ACVR1	SELT
TLR1	PRIM1
EPHB2	IL13RA1
KIFC3	IL2RA
STX2	DRG1
YIPF5	DBI
SMAD7	GNA15
MARCO	FKBP5
FXYD5	PLAUR
CD33	SERPINB9
LMCD1	CEACAM1
RGS3	FLI1
OSTF1	NR3C1
SHC2	ATG3
PRKCB1	SECTM1
PBX3	BTN2A2
GPR176	UBE1L
SLC24A3	C1GALT1C1
PALM	SAT1
BTK	FBXO5

LOC26010	BAK1
TCP11L1	CFLAR
DYNLT3	CD1D
MSX2	BTG3
PPARG	ITGAX
CD34	SLC8A1
GPR137B	PIK3IP1
EPHA2	CCNB2
KCNJ15	RASSF4
GOLIM4	HNMT
CD86	TCN2
PRND	PLA2G4C
SORBS1	NAGK
ADH1A	NFKB1
TFPI	SAR1B
C4ORF31	DDO
SIGLEC9	HAMP
HHEX	CLEC2B
ATP10A	TCIRG1
PSD3	PTPN7
SYNE1	UPP1
KCTD12	FRK
HCFC1R1	SMAD1
MAP7D1	MAN1A1
CCR1	CHRNA6
TXNDC15	LOC93349
OSTM1	CD79A
TPST1	XCL1
AKAP13	LRP8
MCAM	DOCK10
VAMP5	PSMA5
LBH	CD58
TNFRSF9	NAGA
SHOX2	SP110
EN1	ME2
PCDHGC3	LRMP
ROBO1	PARP8
PPP3CA	ALOX5
CHPF	CD28
IL2RA	C17ORF60
ALDH1B1	C1ORF54
MAGEL2	TPST2
PLA2G5	CD300C
IL16	PDCD10
ITGB1	PLEKHQ1
DAAM2	GPSM3

NP	CTSC
C1ORF78	CSF1
KERA	RASGRP3
MFSD1	CD244
RASGRP3	CAPG
PDE4B	GPR18
C1ORF54	GSDML
PLOD1	IRF7
CD37	IL6R
SC65	MELK
SLC39A8	ME1
NRP2	LSP1
TMEM49	DHX58
FKBP11	SLC43A3
ST8SIA4	EMR1
LCP1	CDCP1
GJA7	RFX5
PTGER4	LAT2
GIMAP6	SLC2A5
SLA	LHFPL2
TGFB1	IFITM1
SLC20A1	GRN
S100A3	TRPV2
LHFPL2	ITGB7
DST	SIDT1
PLXND1	PSTPIP1
ATP6V1B2	FGD2
DFNA5	FMNL1
JUNB	P2RX7
APBB2	WIPF1
P4HA1	LYN
PLK3	VAMP5
ELK3	IRF4
SLC16A3	EAF2
ANXA6	P2RY13
SUSD5	SLC35D1
BDKRB2	CSF3R
ANXA5	SAMD9
TEK	ALOX12P2
ASAHL	DCK
MXRA7	PILRA
PPP1R3C	STK17B
MGAT4A	GYG1
STOM	RNF19B
MRAS	LILRB2
CLDN11	FPR1

CKAP4	YWHAH
SLC46A3	PTPRO
ACTC1	NUPR1
PAPPA	IRF9
ENTPD7	FGR
FOXO1	PAMCI
ASPA	ADAM8
IL10	CTSL1
RTN4	PIM2
PTPRG	XBP1
INPP4B	MFSD1
ADAM19	IKZF1
MAN2A1	STAB1
VSNL1	FCN1
ST8SIA1	HERC6
RIN2	SH2B3
F3	IL32
DNAJB4	RAB32
ETS1	C9ORF46
NAP1L3	CTSH
LOH3CR2A	MAD2L1
GUCY1A2	ATP8B4
ROR2	PLEKHF1
RNASE4	IFNGR1
ENTPD1	MX2
IFI30	CD40
CD28	PSTPIP2
INPP5F	JAK2
PTHLH	NLRP3
CENTA2	EBI3
KIAA0247	SDS
CLIC2	BTN3A1
CORO1C	CD5
CCL21	KCNA3
C17ORF60	DEPDC1
IL1RAP	AP1S2
CDR2	CCNA2
CALU	EMP3
FGF18	OPTN
KLHL4	MGAT4A
TSKU	NFKBIE
PGCP	MICB
FHL3	IL18
C13ORF18	DRAM
MEF2C	ICAM1
SCD	ZAP70

ITGA8	TDRD7
CAV2	TNFRSF14
ARHGAP15	OLR1
NRP1	DDX58
CDH5	VAV1
TANC2	PSME1
NLRP3	CD44
EPAS1	KMO
KCNK2	CYBA
IL4R	PARP12
FCGR1A	HTATIP2
MYCT1	C1ORF38
RAMP3	SELPLG
FHOD3	CD300A
TBC1D8B	PSCD4
FZD7	PDE4B
HBEGF	TMEM176B
P2RY14	ARHGEF6
ITGAV	PMAIP1
DOCK2	CBR3
CHRNA1	BTC
DKFZP586H2123	ACSL5
NPL	C21ORF91
TRIB2	CD226
IGSF6	MPP1
MYH10	SOAT1
PPFIBP1	PTPRCAP
A2M	TNFRSF9
CMKLR1	NCK1
MC1R	CD80
FLNA	ABHD3
CDKN1A	MYO1F
NR4A3	P2RY5
HLA-DPA1	KLRD1
CLEC4A	SMPDL3A
CNN3	BLNK
OLFML2A	TBXAS1
SLIT3	P2RY10
CLEC7A	CIITA
CTSS	STAP1
CES1	A2M
SH3PXD2A	CTLA4
MLLT11	TNFRSF1B
SLAMF8	FBP1
PCOLCE2	ITGAM
DUSP5	PYCARD

ARHGAP24	NOD2
ITGA4	NQO1
FAM46A	TAP2
SNED1	CLEC5A
CD52	PVRIG
HCLS1	MOCOS
TLR7	PTAFR
FER1L3	CYP2J2
MSN	STAT4
AOAH	LIPA
RCAN1	CST7
LRP12	NMI
PFTK1	IL7
HSPB8	HMHA1
CD302	TLR6
PTPRE	RNASE2
PARVA	SELL
SKAP2	EPB41L3
MYH11	PYHIN1
CLEC5A	LILRB3
FOXF2	NXT2
PDCD1LG2	CDKN2A
PTGS2	AMPD1
MAP4K4	ITGAL
ARHGAP28	CD84
CD48	ANXA10
SCHIP1	ITGA4
ADAM9	TRIM21
LAIR1	DHRS3
SNX10	CRTAM
GALNACT-2	TAPBPL
KDR	KLRC3
PHLDA1	IRAK3
EDG1	PIK3CG
C11ORF75	LILRB4
STK17B	C1S
LEPREL2	CHI3L1
DARC	CD180
C3ORF64	ISG15
IL10RA	TLR3
OSMR	CCR6
GAS7	NR1H3
BMP1	TNIP3
NCKAP1L	RAB27A
MDFIC	FAM129A
HCK	CD4

CTSO	SP140
TNFRSF12A	IFIT2
C5ORF13	APOBEC3B
BCL2A1	B2M
BDKRB1	LCK
PSCD3	SERPINB1
FSCN1	IFI16
IGFBP3	HLA-G
LRRC32	KLRK1
FEZ1	LAX1
GPR65	CD96
RGS17	IL21R
TNN	ITK
APBB1IP	VNN3
SLC31A2	BTN3A3
ACTN1	SIRPG
C11ORF41	STXBP6
PROCR	PTPN22
RECK	CD37
TNFAIP3	CTSW
ANXA2	APOC2
PTGIR	SIGLEC1
APOD	SLCO2B1
SELP	USP18
ST3GAL1	PSME2
PRPH2	MX1
BNC2	SERPINA1
FOLR2	FPRL1
MFGE8	IKZF3
SEPT8	CYB5R4
TLR2	RNASE1
PDLIM5	IL1B
KHDRBS3	RAC2
MAF	SAMHD1
NCF2	FAM105A
PTGER2	TLR4
HLA-DPB1	KYNU
CLSTN2	ACP5
HMOX1	SLC31A2
HEYL	HMOX1
AKAP12	IRF8
CHSY1	LGALS2
CLIC4	CECR1
RND3	CLIC2
FBXL7	BTN3A2
IL32	BIN2

TMEM47	LGALS9
IER3	GCH1
SPSB1	CSTA
FPR1	TRAF3IP3
FLI1	DAPP1
GBP2	NPL
CCR7	ISG20
ATP10D	MGC29506
PDGFC	CD72
PVALB	CSF1R
SEC24D	CCNE1
DLC1	CD33
ARSJ	IL7R
FGL2	OAS3
MYH9	SIGLEC9
HAPLN1	TMEM156
EMCN	PRF1
SH2B3	TLR1
CSF1R	APOE
TGFBR2	ST8SIA4
PLEKHC1	IFI6
AGTRL1	LY75
GGTLA1	CP
ELN	TMEM149
GLRX	SLC15A3
GREM2	HLA-DOA
LCP2	APOL1
GALNT10	SLA
AXL	TLR7
ANXA1	CENTA2
FYB	HLA-A
SKIL	BCL2A1
KRT17	CFB
GJA5	IL15RA
ENC1	ZBP1
MSR1	LAP3
SPRY4	HLA-DOB
FLT1	MSR1
IRAK3	ECGF1
VEGFC	CXCR3
LY86	SPP1
TIMP2	LCN2
RASSF2	CD3E
ITGB5	DOCK2
ABCA1	CCR1
RBMS3	GPNMB

HS3ST3A1	IFI27
SYTL2	TMEM140
P4HA2	SNX10
RUNX1	LAIR2
APOC1	LCP1
PKD2	ADORA3
CCDC102B	LILRB1
AVPR1A	UBE2L6
ZNF365	STAT1
TPM1	PDCD1LG2
BMP2	PLSCR1
CUGBP2	NCF4
NEDD4	SH2D1A
ARHGEF6	OAS2
ENPEP	LAIR1
MYL9	PSMB10
MMP3	CCDC109B
HSPB7	HPSE
CD2	GLRX
EIF5A2	HOXD1
SLCO2B1	TNFAIP8
PRDM1	NCF1
HLX	CCL2
AIF1	RHOH
WIPF1	PLAC8
MAN1A1	IFIT1
EHD2	ICOS
PLEK	TRAT1
P2RY5	TREM2
LIF	CXCR6
TPM2	CCR7
ANGPT1	FCGR1A
DCLK1	ARHGDIB
OSR2	GIMAP6
AP1S2	BIRC3
KLF10	XAF1
TUBB2A	BTK
EFEMP2	APOBEC3A
SLC7A7	GFI1
IGFBP6	CMKLR1
SEC23A	SOD2
PDLIM2	CORO1A
TRIM22	EBI2
RNASE6	LY96
FLNC	FLJ20035
SGK	UBASH3A

MALL	OAS1
KLF6	RSAD2
VDR	WARS
ARHGDIB	LPXN
PID1	POU2AF1
MMD	CD27
DRAM	IFIH1
SEMA5A	CLEC7A
HSPB3	LST1
PRKG1	GIMAP5
AFAP1	FCGR2A
TLR4	CCR5
ZFP36	IFI44
DPP4	EVI2A
HTR2B	HLA-E
CALCRL	AOAH
DCHS1	HCLS1
MEOX2	CHODL
KCNJ8	VTCN1
FAS	ARHGAP15
PPIC	IL18RAP
CAV1	NCF2
AHNAK2	BATF
ISL1	IL10RA
ID1	HLA-F
ZMAT3	APOL6
TREM1	C2
FOXF1	CXORF21
DOCK4	GBP2
PLOD2	DPYD
RGS5	FPRL2
TYROBP	LAMP3
SGCD	IL2RB
CMAH	APBB1IP
PTGER3	GNLY
FSTL3	CD86
C7ORF10	SLC7A7
C1QTNF1	LY86
AKT3	SLAMF1
FBLN5	CD14
SAMSN1	TLR2
LOC728215	FYB
ADAMTSL3	HCK
CLIC3	HLA-C
PDZRN3	PSMB8
C5AR1	PDZK1IP1

GPR116	APOL3
SLC16A7	CXORF9
SCUBE2	IFI44L
EPB41L3	OASL
ICAM1	SQRDL
MN1	KLRB1
PROS1	IRF1
KLK6	AIF1
CHST11	CLEC4A
CXCR7	GMFG
ADAMTS9	ITGB2
CXORF21	ETV7
DSC3	IFI35
LRP1	LTB
GIMAP4	CCR2
ST6GALNAC5	IL15
TGM2	IGSF6
SEPT11	HLA-B
SMPDL3A	RTP4
ELL2	GPR171
GMFG	SLAMF8
TCF4	RGS1
TLR8	RARRES3
ADAMTS2	GZMK
NAV3	HERC5
OLR1	CTA-246H3.1
MRC2	NCKAP1L
PTGDS	GBP1
MYO1B	C6ORF105
ADM	TRIM22
EMP3	CD247
ABCA6	TNFSF10
MME	HCP5
COX7A1	PLEK
S100A10	C3AR1
SERPINH1	VSIG4
CPZ	APOBEC3G
PSCDBP	CD3D
LEPRE1	VNN2
PODNL1	PSCDBP
MYLK	CD163
GFRA1	IGLV2-14
MGP	CD3G
BARX1	HLA-DMB
PTPRD	LCP2
CD69	GZMB

MGC14376	CCL7
C13ORF15	MNDA
FCER1G	NKG7
MICAL2	SAMSN1
LSAMP	CD52
C20ORF103	IFI30
PRKCDBP	TNFRSF17
XYLT1	FGL2
FPRL2	IGHM
IGFBP7	CASP1
CACNA1C	APOC1
EGR1	GIMAP4
MMP19	RNASE6
C16ORF30	GPR65
ABCA8	SRGN
TUBB6	TLR8
GLI3	TFEC
KRT6A	CD69
FOSB	MMP9
C1QA	IL2RG
FLRT2	GZMH
MARCKS	FCGR2B
CCL18	TYROBP
ZFPM2	CD74
LMOD1	EVI2B
BHLHB2	FCER1G
KDELC1	MS4A6A
LIMA1	LAPTM5
PPAP2A	C1QB
MNDA	HLA-DRA
C3	HLA-DMA
EDG2	TAP1
ITGB2	MS4A4A
C1QB	HLA-DPA1
PRSS23	PTPRC
EMILIN1	ALOX5AP
DPYD	HLA-DPB1
KCND2	CTSS
ITGB6	C1QA
FMO2	SIT1
CREB3L1	LAG3
CYBB	IFNG
SRGN	CD48
GPR124	CYBB
CPE	SLAMF7
IGHM	CD53

PLXNC1
PLAUR
TPBG
PLVAP
ZEB2
GADD45B
CST7
KIAA1199
HOM-TES-103
RAI14
DKK2
GPR87
THBD
JAM3
FCGR2B
G0S2
KAL1
ID3
LGI2
RHOBTB3
NKX3-2
UPK1B
GHR
FBLN1
SELE
GNG11
ENOX1
SDC2
FAT
CFD
COL4A2
HOXA5
LTBP2
TMEM176A
CCL2
GZMK
PCDH17
PLAT
SDC1
C3AR1
CD14
SLC2A3
MS4A6A
PDE10A
MSC
FZD1

CCL5
CLEC4E
CD38
CD2
INDO
PSMB9
AIM2
CXCL13
CXCL10
IGKC
PIGR
LYZ
CXCL11
IGKV1-5
UBD
CXCL9
ADAMDEC1

TMEM176B
KLF2
WNT7A
MGC4294
COL15A1
SPARCL1
TMEM2
FCGR2A
ALOX5AP
GALNAC4S-6ST
VIM
EGFL6
LAPTM5
EVI2B
NDN
LDB2
DUSP1
ODZ3
AMIGO2
CORIN
KDELR3
RCAN2
FKBP14
VGLL3
PLIN
CD93
SPHK1
GPR1
GAS1
CD53
FOS
NID1
VSIG4
PTPRC
BMP4
EMP1
LXN
PRKD1
CNTN1
TGFB3
SLIT2
ABCC9
ACSL1
DSE
MAFB
MATN3

PCSK5
KIF26B
SCRG1
CYP26A1
CLDN16
GPR23
C10ORF56
AGTR1
SPP1
CHN1
IL6
SLC12A8
NCAM2
LAMA4
EGR2
COL6A1
ELTD1
EBI2
SNAI1
DDR2
JAM2
LOXL2
MMP14
ZFHX4
PAPSS2
PXDN
ENPP1
HAS2
PTRF
LY96
RUNX2
LPL
COL8A2
CNN2
SPOCK1
MFAP2
IGF1
ETV1
BNC1
CD163
CD248
AQP1
COL4A1
DAB2
HEG1
PDGFRL

LYZ
LGALS1
FBLN2
SEMA3C
MS4A4A
AK5
TGFB1I1
ITGA5
DPYSL3
RGS1
TNFSF4
MFAP4
ZEB1
BCHE
CH25H
ANTXR1
WISP1
IGFBP4
ISLR
PLK2
PDGFRB
TNC
AOC3
ADAMTS5
CYP7B1
LHFP
LZTS1
CNN1
CALD1
BASP1
MXRA8
HSD17B6
HTRA1
NOX4
EVI2A
TMEM45A
PDGFD
THY1
LRRC17
IGKC
EFEMP1
RARRES2
PLN
CYP1B1
RGS16
PDGFRA

OGN
RUNX1T1
CD36
RGS4
ANGPTL2
RGS2
OLFML1
RCN3
PMP22
PALLD
PLS3
FGF1
HOPX
DACT1
CFH
C1S
MXRA5
GFPT2
C4ORF18
CTGF
ADH1B
OLFML3
PLXDC1
NBL1
F2R
KCNE4
COL5A3
FSTL1
LOXL1
FAM38B
ADIPOQ
CPA3
CLEC2B
TMEPAI
ADRA2A
EDNRA
GEM
DIO2
FILIP1L
SRPX2
SVEP1
LAMB1
CCRL1
CYR61
PPEF1
BGN

SPON2
GJA1
MMP1
ALDH1A3
MOXD1
NID2
GPNMB
DPT
PDPN
GUCY1A3
RAB31
NUAK1
COL6A2
CILP
ADAMTS12
SEMA3D
IL7R
TGFBI
ECM1
PTGIS
CALB2
HEPH
CDR1
FGF7
OLFML2B
NT5E
COL16A1
SRPX
COL8A1
PRRX1
PITX2
F13A1
SCG2
PCOLCE
PDLIM3
TAGLN
THBS1
TWIST1
ECM2
OMD
COPZ2
MFAP5
LOX
C1QTNF3
SERPINE1
ITGBL1

TNFAIP6
LPPR4
HNT
GLT8D2
ADAM12
SPARC
TMEM158
ACTG2
TIMP3
CCL11
FMO1
CXCL12
ACTA2
EDIL3
CRISPLD2
NNMT
GREM1
COLEC12
FN1
FBN1
COL1A2
SNAI2
AEBP1
PLAU
TDO2
VCAM1
SULF1
COL6A3
MMP2
COL5A1
SERPINF1
COL3A1
LRRC15
CDH11
MMP11
SFRP4
COL1A1
DCN
INHBA
CXCL14
EPYC
GRP
FABP4
COMP
VCAN
CTSK

COL10A1
LUM
MMP13
FAP
ASPN
COL5A2
THBS2
COL11A1
POSTN

Supplementary Table S3
Transcription factors used in the network analysis

No	**Transcription Factor**
1	AATF
2	ADNP
3	AEBP1
4	AFF1
5	AFF3
6	AFF4
7	AHCTF1
8	AHR
9	ALX4
10	AR
11	ARID3A
12	ARID4A
13	ARNT
14	ARNT2
15	ARNTL
16	ARNTL2
17	ASCL1
18	ASCL2
19	ATBF1
20	ATF1
21	ATF2
22	ATF3
23	ATF4
24	ATF5
25	ATF6
26	ATF7
27	ATOH1
28	BACH1
29	BACH2
30	BAPX1
31	BARX2
32	BATF
33	BAZ1B
34	BCL6
35	BHLHB2
36	BHLHB3
37	BLZF1
38	BNC1
39	BRD8
40	BRF1

41	BRPF1
42	BTAF1
43	BUD31
44	C2orf3
45	CBFA2T2
46	CBFA2T3
47	CBFB
48	CBL
49	CCRN4L
50	CDX1
51	CDX2
52	CDX4
53	CEBPA
54	CEBPB
55	CEBPD
56	CEBPE
57	CEBPG
58	CEBPZ
59	CHES1
60	CIITA
61	CIR
62	CITED1
63	CITED2
64	CLOCK
65	CNBP
66	CNOT7
67	CNOT8
68	CREB1
69	CREB3
70	CREB3L1
71	CREB3L2
72	CREB5
73	CREBBP
74	CREBL1
75	CREBL2
76	CREG1
77	CREM
78	CRX
79	CSDA
80	CTBP1
81	CTBP2
82	CTCF
83	CTNNB1
84	CUTL1
85	CUTL2
86	DAXX

87	DBP
88	DDIT3
89	DEK
90	DENND4A
91	DLX2
92	DLX4
93	DLX5
94	DLX6
95	DMTF1
96	DR1
97	DRAP1
98	DSCR1
99	DUX1
100	E2F1
101	E2F2
102	E2F3
103	E2F4
104	E2F5
105	E2F6
106	E2F8
107	E4F1
108	EDF1
109	EGR1
110	EGR2
111	EGR3
112	EGR4
113	ELF1
114	ELF2
115	ELF3
116	ELF4
117	ELF5
118	ELK1
119	ELK3
120	ELK4
121	EMX1
122	EMX2
123	EN1
124	EN2
125	ENO1
126	EP300
127	EPAS1
128	ERCC6
129	ERF
130	ERG
131	ESR1
132	ESR2

133	ESRRA
134	ESRRB
135	ESRRG
136	ETS1
137	ETS2
138	ETV1
139	ETV3
140	ETV4
141	ETV5
142	ETV6
143	ETV7
144	EVI1
145	EVX1
146	EWSR1
147	FALZ
148	FEV
149	FEZF2
150	FLI1
151	FMNL2
152	FOS
153	FOSB
154	FOSL1
155	FOSL2
156	FOXA1
157	FOXA2
158	FOXB1
159	FOXD1
160	FOXD3
161	FOXE1
162	FOXE3
163	FOXF1
164	FOXF2
165	FOXG1B
166	FOXH1
167	FOXI1
168	FOXJ1
169	FOXJ2
170	FOXJ3
171	FOXK2
172	FOXL1
173	FOXM1
174	FOXN1
175	FOXO1A
176	FOXO3A
177	FOXP1
178	FOXP3

179	FUBP1
180	FUBP3
181	GABPB2
182	GAS7
183	GATA1
184	GATA2
185	GATA3
186	GATA4
187	GATA6
188	GATAD1
189	GATAD2A
190	GBX2
191	GLI2
192	GLI3
193	GMEB1
194	GRLF1
195	GTF2IRD1
196	HAND1
197	HAND2
198	HBP1
199	HCFC1
200	HCLS1
201	HES1
202	HES2
203	HESX1
204	HEY1
205	HEY2
206	HEYL
207	HHEX
208	HIC1
209	HIF1A
210	HIF3A
211	HIRA
212	HIVEP1
213	HIVEP2
214	HIVEP3
215	HKR3
216	HLF
217	HLX1
218	HLXB9
219	HMBOX1
220	HMG20A
221	HMG20B
222	HMGA1
223	HMGA2
224	HMGB1

225	HMGB2
226	HMX1
227	HNF4A
228	HNF4G
229	HOP
230	HOXA1
231	HOXA10
232	HOXA11
233	HOXA2
234	HOXA3
235	HOXA4
236	HOXA5
237	HOXA6
238	HOXA7
239	HOXA9
240	HOXB13
241	HOXB2
242	HOXB5
243	HOXB6
244	HOXB7
245	HOXB8
246	HOXB9
247	HOXC10
248	HOXC11
249	HOXC4
250	HOXC5
251	HOXC6
252	HOXD1
253	HOXD10
254	HOXD11
255	HOXD12
256	HOXD13
257	HOXD3
258	HOXD4
259	HOXD9
260	H-plk
261	HR
262	HSF1
263	HSF2
264	HSF4
265	HTLF
266	IKZF1
267	IKZF4
268	IKZF5
269	ILF2
270	INSM1

271	IPF1
272	IRF1
273	IRF2
274	IRF3
275	IRF4
276	IRF5
277	IRF6
278	IRF7
279	IRF8
280	IRX4
281	IRX5
282	ISGF3G
283	ISL1
284	JARID1A
285	JARID1B
286	JUN
287	JUNB
288	JUND
289	KIAA0415
290	KIAA0963
291	KLF1
292	KLF10
293	KLF11
294	KLF12
295	KLF13
296	KLF15
297	KLF2
298	KLF3
299	KLF4
300	KLF5
301	KLF6
302	KLF7
303	KLF9
304	KNTC1
305	L3MBTL
306	LASS2
307	LASS4
308	LASS6
309	LBX1
310	LHX2
311	LHX3
312	LHX5
313	LHX6
314	LMO1
315	LMO4
316	LMX1B

317	LOC645682
318	LYL1
319	LZTFL1
320	LZTR1
321	LZTS1
322	MAF
323	MAFB
324	MAFF
325	MAFG
326	MAFK
327	MAML3
328	MAX
329	MAZ
330	MBD1
331	MDS1
332	MECP2
333	MEF2A
334	MEF2B
335	MEF2C
336	MEF2D
337	MEIS1
338	MEIS2
339	MEIS3P1
340	MEOX1
341	MEOX2
342	MGA
343	MITF
344	MIZF
345	MLL
346	MLL4
347	MLLT10
348	MLLT7
349	MLX
350	MLXIP
351	MLXIPL
352	MNT
353	MSC
354	MSL3L1
355	MSRB2
356	MSX1
357	MSX2
358	MTA1
359	MTA2
360	MTF1
361	MXD1
362	MYB

363	MYBL1
364	MYBL2
365	MYC
366	MYCL1
367	MYCN
368	MYF6
369	MYNN
370	MYOD1
371	MYOG
372	MYST2
373	MYT1
374	MYT1L
375	MZF1
376	NANOG
377	NCOR1
378	NEUROD1
379	NEUROD2
380	NEUROG1
381	NEUROG3
382	NFAT5
383	NFATC1
384	NFATC3
385	NFATC4
386	NFE2
387	NFE2L1
388	NFE2L2
389	NFE2L3
390	NFIB
391	NFIC
392	NFIL3
393	NFIX
394	NFKB1
395	NFKB2
396	NFRKB
397	NFX1
398	NFYA
399	NFYB
400	NFYC
401	NHLH1
402	NHLH2
403	NKRF
404	NKX2-2
405	NKX2-5
406	NKX2-8
407	NKX3-1
408	NKX6-1

409	NOTCH2
410	NPAS2
411	NPAS3
412	NPAT
413	NR0B1
414	NR0B2
415	NR1D2
416	NR1H2
417	NR1H3
418	NR1H4
419	NR1I2
420	NR1I3
421	NR2C1
422	NR2C2
423	NR2E1
424	NR2E3
425	NR2F1
426	NR2F2
427	NR2F6
428	NR3C1
429	NR3C2
430	NR4A1
431	NR4A2
432	NR4A3
433	NR5A1
434	NR5A2
435	NR6A1
436	NRF1
437	NRL
438	OLIG2
439	ONECUT1
440	OVOL1
441	PAX1
442	PAX2
443	PAX3
444	PAX4
445	PAX6
446	PAX7
447	PAX8
448	PAX9
449	PBX1
450	PBX2
451	PBX3
452	PCGF2
453	PEG3
454	PFDN1

455	PGR
456	PHF2
457	PHOX2A
458	PHOX2B
459	PHTF1
460	PHTF2
461	PITX1
462	PITX3
463	PKNOX1
464	PKNOX2
465	PLAG1
466	PLAGL1
467	PLAGL2
468	PML
469	POU2F1
470	POU2F2
471	POU2F3
472	POU3F1
473	POU3F2
474	POU3F3
475	POU3F4
476	POU4F1
477	POU4F2
478	POU6F1
479	POU6F2
480	PPARA
481	PPARD
482	PPARG
483	PRDM1
484	PRDM16
485	PRDM2
486	PREB
487	PROP1
488	PRRX1
489	PRRX2
490	PTTG1
491	PURA
492	RARA
493	RARB
494	RARG
495	RAX
496	RB1
497	RBL2
498	RBPSUH
499	RBPSUHL
500	REL

501	RELA
502	RELB
503	RERE
504	REST
505	REXO4
506	RFX1
507	RFX2
508	RFX3
509	RFX5
510	RFXANK
511	RFXAP
512	RLF
513	RNF4
514	RORA
515	RORB
516	RORC
517	RREB1
518	RUNX1
519	RUNX1T1
520	RUNX2
521	RUNX3
522	RXRA
523	RXRB
524	RXRG
525	SALL1
526	SALL2
527	SATB1
528	SATB2
529	SCAND1
530	SCAND2
531	SCML1
532	SCML2
533	SHOX
534	SHOX2
535	SIM2
536	SIX1
537	SIX2
538	SIX3
539	SIX5
540	SIX6
541	SLC26A3
542	SLC2A4RG
543	SLC30A9
544	SMAD1
545	SMAD2
546	SMAD3

547	SMAD4
548	SMAD5
549	SMAD6
550	SMAD7
551	SMAD9
552	SMARCA3
553	SMARCA4
554	SNAI1
555	SNAI2
556	SNAPC2
557	SNAPC4
558	SNAPC5
559	SNFT
560	SOLH
561	SOX1
562	SOX10
563	SOX11
564	SOX12
565	SOX13
566	SOX15
567	SOX17
568	SOX18
569	SOX2
570	SOX21
571	SOX3
572	SOX4
573	SOX5
574	SOX9
575	SP1
576	SP140
577	SP2
578	SP3
579	SP4
580	SPDEF
581	SPI1
582	SPIB
583	SREBF1
584	SREBF2
585	SRF
586	ST18
587	STAT1
588	STAT2
589	STAT3
590	STAT4
591	STAT5A
592	STAT5B

593	STAT6
594	SUPT4H1
595	SUPT6H
596	T
597	TADA2L
598	TADA3L
599	TAF1B
600	TAF5L
601	TAL1
602	TARDBP
603	TBR1
604	TBX1
605	TBX10
606	TBX19
607	TBX2
608	TBX21
609	TBX3
610	TBX4
611	TBX5
612	TBX6
613	TCEAL1
614	TCF1
615	TCF12
616	TCF15
617	TCF2
618	TCF21
619	TCF25
620	TCF3
621	TCF4
622	TCF7
623	TCF7L1
624	TCF7L2
625	TCF8
626	TCFL5
627	TEAD1
628	TEAD3
629	TEAD4
630	TEF
631	TFAM
632	TFAP2A
633	TFAP2B
634	TFAP2C
635	TFAP4
636	TFCP2
637	TFCP2L1
638	TFDP1

639	TFDP2
640	TFDP3
641	TFE3
642	TFEB
643	TFEC
644	TGIF
645	TGIF2
646	THRA
647	THRB
648	TLX1
649	TLX2
650	TNRC4
651	TP53
652	TP73
653	TP73L
654	TRERF1
655	TRIM22
656	TRIM25
657	TRIM28
658	TRIM29
659	TRPS1
660	TSC22D1
661	TSC22D2
662	TSC22D3
663	TSC22D4
664	TULP4
665	TWIST1
666	UBN1
667	UBP1
668	USF2
669	VAV1
670	VAX2
671	VDR
672	VENTX
673	VEZF1
674	VPS72
675	VSX1
676	WT1
677	XBP1
678	YBX1
679	YEATS4
680	YWHAE
681	YWHAZ
682	YY1
683	YY2
684	ZBTB16

685	ZBTB17
686	ZBTB22
687	ZBTB25
688	ZBTB38
689	ZBTB43
690	ZBTB6
691	ZBTB7A
692	ZBTB7B
693	ZF
694	ZFHX1B
695	ZFHX4
696	ZFP36L1
697	ZFP36L2
698	ZFP37
699	ZFP95
700	ZFX
701	ZFY
702	ZHX2
703	ZHX3
704	ZIC1
705	ZIM2
706	ZKSCAN1
707	ZMYM2
708	ZMYM3
709	ZMYM4
710	ZNF10
711	ZNF117
712	ZNF12
713	ZNF124
714	ZNF131
715	ZNF132
716	ZNF133
717	ZNF134
718	ZNF135
719	ZNF136
720	ZNF137
721	ZNF14
722	ZNF140
723	ZNF141
724	ZNF142
725	ZNF143
726	ZNF146
727	ZNF148
728	ZNF154
729	ZNF155
730	ZNF16

731	ZNF160
732	ZNF167
733	ZNF174
734	ZNF175
735	ZNF177
736	ZNF180
737	ZNF184
738	ZNF185
739	ZNF187
740	ZNF189
741	ZNF192
742	ZNF193
743	ZNF195
744	ZNF197
745	ZNF20
746	ZNF200
747	ZNF202
748	ZNF204
749	ZNF205
750	ZNF207
751	ZNF211
752	ZNF212
753	ZNF215
754	ZNF217
755	ZNF219
756	ZNF22
757	ZNF221
758	ZNF222
759	ZNF223
760	ZNF224
761	ZNF225
762	ZNF226
763	ZNF227
764	ZNF228
765	ZNF230
766	ZNF232
767	ZNF235
768	ZNF236
769	ZNF238
770	ZNF239
771	ZNF24
772	ZNF248
773	ZNF250
774	ZNF253
775	ZNF259
776	ZNF26

777	ZNF263
778	ZNF264
779	ZNF266
780	ZNF267
781	ZNF268
782	ZNF271
783	ZNF273
784	ZNF274
785	ZNF277
786	ZNF278
787	ZNF281
788	ZNF282
789	ZNF286
790	ZNF287
791	ZNF289
792	ZNF291
793	ZNF292
794	ZNF294
795	ZNF3
796	ZNF302
797	ZNF304
798	ZNF306
799	ZNF307
800	ZNF313
801	ZNF318
802	ZNF32
803	ZNF322B
804	ZNF323
805	ZNF324
806	ZNF329
807	ZNF330
808	ZNF331
809	ZNF334
810	ZNF335
811	ZNF337
812	ZNF33B
813	ZNF34
814	ZNF343
815	ZNF345
816	ZNF35
817	ZNF350
818	ZNF354A
819	ZNF358
820	ZNF364
821	ZNF365
822	ZNF384

823	ZNF394
824	ZNF395
825	ZNF403
826	ZNF407
827	ZNF408
828	ZNF409
829	ZNF410
830	ZNF415
831	ZNF419A
832	ZNF42
833	ZNF423
834	ZNF426
835	ZNF43
836	ZNF430
837	ZNF432
838	ZNF434
839	ZNF435
840	ZNF44
841	ZNF440
842	ZNF443
843	ZNF444
844	ZNF446
845	ZNF447
846	ZNF45
847	ZNF451
848	ZNF460
849	ZNF467
850	ZNF468
851	ZNF471
852	ZNF473
853	ZNF480
854	ZNF484
855	ZNF493
856	ZNF500
857	ZNF506
858	ZNF507
859	ZNF508
860	ZNF510
861	ZNF516
862	ZNF518
863	ZNF528
864	ZNF529
865	ZNF532
866	ZNF536
867	ZNF544
868	ZNF549

869	ZNF550
870	ZNF551
871	ZNF552
872	ZNF556
873	ZNF557
874	ZNF562
875	ZNF573
876	ZNF574
877	ZNF576
878	ZNF580
879	ZNF586
880	ZNF587
881	ZNF588
882	ZNF589
883	ZNF592
884	ZNF593
885	ZNF606
886	ZNF609
887	ZNF611
888	ZNF614
889	ZNF623
890	ZNF629
891	ZNF638
892	ZNF643
893	ZNF646
894	ZNF652
895	ZNF654
896	ZNF659
897	ZNF665
898	ZNF667
899	ZNF668
900	ZNF669
901	ZNF671
902	ZNF672
903	ZNF673
904	ZNF675
905	ZNF682
906	ZNF688
907	ZNF692
908	ZNF695
909	ZNF696
910	ZNF7
911	ZNF701
912	ZNF702
913	ZNF706
914	ZNF710

915	ZNF711
916	ZNF74
917	ZNF75
918	ZNF79
919	ZNF8
920	ZNF81
921	ZNF83
922	ZNF84
923	ZNF85
924	ZNF91
925	ZNF93
926	ZNF96
927	ZNFN1A1
928	ZSCAN5
929	SCMH1
930	ZBTB32
931	DPEP1
932	WWTR1
933	HOXC8
934	MED17
935	FOXN3
936	FOXC1
937	GSC2
938	TAF2
939	POU1F1
940	TAF11
941	HLTF
942	TP63
943	ZNF37A
944	GTF3C1
945	GTF2I
946	UBE2K
947	AKR1B1
948	RRN3
949	KCNH4
950	POU4F3
951	GMEB2
952	ADNP2
953	HTATSF1
954	TAF7L
955	TAF1C
956	GABPB1
957	BRF2
958	RNASEH2A
959	ETV2
960	GARS

961	PDLIM1
962	UBTF
963	ACCN1
964	HNF1B
965	NKX3_2
966	HTATIP2
967	PDHX
968	PRDM4
969	CYP27B1
970	MYF5
971	CUX2
972	HCFC2
973	BTN3A3
974	SIM1
975	MED23
976	TBP
977	SUB1
978	TCERG1
979	GTF3C2
980	HDAC1
981	ASH1L
982	IVNS1ABP
983	PROX1
984	CD3EAP
985	FOXO3
986	ELL2
987	GTF3C3
988	ONECUT2
989	HOXB3
990	HOXB1
991	KCNJ5
992	TAF12
993	ETF1
994	ZSCAN18
995	GTF3A
996	HOXC13
997	BATF3
998	RBPJL
999	C11orf9
1000	GTF3C4
1001	GTF2F1
1002	NR1D1
1003	PKMYT1
1004	CSRP3
1005	MLLT1
1006	MNX1

1007	TAF4
1008	NPAS1
1009	BARX1
1010	ZSCAN5A
1011	LHX1
1012	HSD17B4
1013	MED6
1014	EIF2S1
1015	HNF1A
1016	EHF
1017	NKX2_1
1018	BRIP1
1019	HLX
1020	DMRT1
1021	KIAA0319
1022	GCM1
1023	CREBZF
1024	YAP1
1025	GTF2B
1026	ALS2CR8
1027	LEF1
1028	PDX1
1029	GTF2A2
1030	ZFHX3
1031	NOL3
1032	TAF4B
1033	TAF1A
1034	TMF1
1035	GC
1036	BTF3
1037	ABT1
1038	ZNF41
1039	TAF1
1040	SLC25A37
1041	GTF3C5
1042	ZEB1
1043	ANKRD1
1044	TAF5
1045	FOXO1
1046	TIAL1
1047	MED21
1048	SMARCA5
1049	GTF2E1
1050	MIA3
1051	GABPA
1052	MED7

1053	ALX3
1054	ZSCAN12
1055	RCAN1
1056	AIRE
1057	PMF1
1058	CUX1
1059	IKZF3
1060	TAF6L
1061	MFI2
1062	PITX2
1063	TLX3
1064	GTF2A1
1065	GFI1B
1066	TAF10
1067	MLL2
1068	SF1
1069	RBPJ
1070	GDNF
1071	SOX14
1072	ZEB2
1073	ZFPM2
1074	PA2G4
1075	SCRT1
1076	FOXN2
1077	HOPX
1078	TCEA2
1079	UTF1
1080	ZNF80
1081	ASCL3
1082	FOXG1
1083	ZSCAN2
1084	FOXC2
1085	TGIF1
1086	TAF7
1087	MGAM
1088	ALX1
1089	FOXO4
1090	SRY
1091	L3MBTL1
1092	FOXD2
1093	TAF9B
1094	ZKSCAN4
1095	GTF2F2
1096	LITAF
1097	ZKSCAN3
1098	SUPT3H

1099	HDAC2
1100	ZKSCAN5
1101	ZSCAN16
1102	GTF2E2
1103	ZNF165
1104	HNRNPAB
1105	TAF13
1106	NCOA6
1107	ZBTB48
1108	GTF2H4
1109	IRF9
1110	EBF2
1111	PSG1

Supplementary Table S4
6 Mesenchymal-specific regulons

Regulon	Universe.Size	Regulon.Size	Total.Hits	Expected.Hits	Observed.Hits	Pvalue	Adjusted.Pvalue
AEBP1	11864	116	1363	13.33	116	0	0
PRRX1	11864	58	1363	6.66	58	0	0
SNAI2	11864	98	1363	11.26	98	0	0
ZEB1	11864	26	1363	2.99	26	0	0
ZEB2	11864	70	1363	8.04	70	0	0
HOPX	11864	41	1363	4.71	41	0	0

Supplementary Table S5

10 Immunoreactive-specific regulons

Regulon	Universe.Size	Regulon.Size	Total.Hits	Expected.Hits	Observed.Hits	Pvalue	Adjusted.Pvalue
BATF	11864	76	1267	8.12	76	0	0
ETV7	11864	35	1267	3.74	35	0	0
HCLS1	11864	49	1267	5.23	49	0	0
IKZF1	11864	93	1267	9.93	93	0	0
IRF7	11864	26	1267	2.78	26	0	0
SP140	11864	54	1267	5.77	54	0	0
TFEC	11864	206	1267	22	206	0	0
BTN3A3	11864	31	1267	3.31	31	0	0
IKZF3	11864	24	1267	2.56	24	0	0
IRF9	11864	22	1267	2.35	22	0	0
